# Block Chemistry for Accurate Modeling of Epoxy Resins

**DOI:** 10.1021/acs.jpcb.3c04724

**Published:** 2023-08-24

**Authors:** Mattia Livraghi, Sampanna Pahi, Piotr Nowakowski, David M. Smith, Christian R. Wick, Ana-Sunčana Smith

**Affiliations:** † Friedrich-Alexander-Universität Erlangen-Nürnberg (FAU), Institute for Theoretical Physics, PULS Group, Interdisciplinary Center for Nanostructured Films (IZNF), Cauerstrasse 3, Erlangen 91058, Germany; ‡ Group for Computational Life Sciences, Division of Physical Chemistry, Ruđer Bošković Institute, Bijenička cesta 54, Zagreb 10000, Croatia

## Abstract

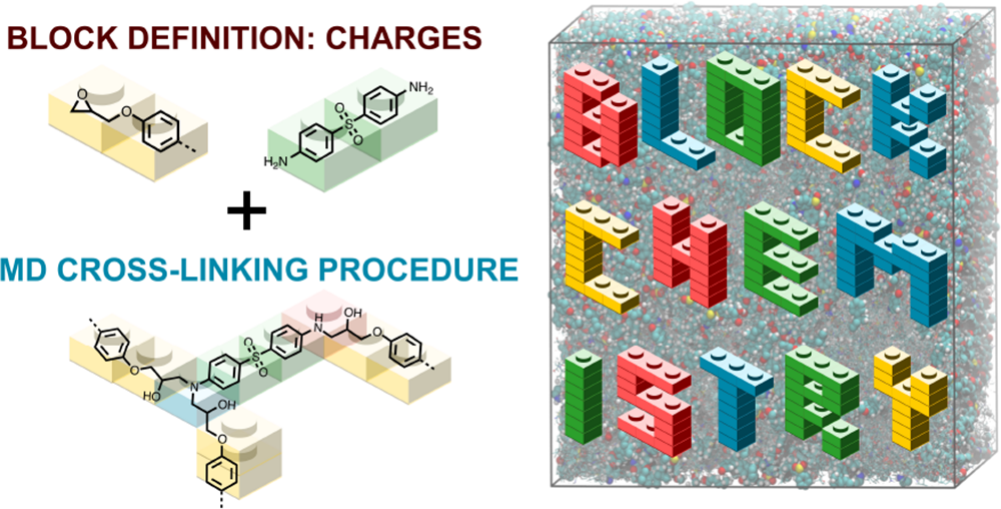

Accurate molecular modeling of the physical and chemical behavior of highly cross-linked epoxy resins at the atomistic scale is important for the design of new property-optimized materials. However, a systematic approach to parametrizing and characterizing these systems in molecular dynamics is missing. We therefore present a unified scheme to derive atomic charges for amine-based epoxy resins, in agreement with the AMBER force field, based on defining reactive fragments—blocks—building the network. The approach is applicable to all stages of curing from pure liquid to gelation to fully cured glass. We utilize this approach to study DGEBA/DDS epoxy systems, incorporating dynamic topology changes into atomistic molecular dynamics simulations of the curing reaction with 127,000 atoms. We study size effects in our simulations and predict the gel point utilizing a rigorous percolation theory to recover accurately the experimental data. Furthermore, we observe excellent agreement between the estimated and the experimentally determined glass transition temperatures as a function of curing rate. Finally, we demonstrate the quality of our model by the prediction of the elastic modulus based on uniaxial tensile tests. The presented scheme paves the way for a broadly consistent approach for modeling and characterizing all amine-based epoxy resins.

## Introduction

Epoxy-resin thermosets are known for their tunable and superior thermomechanical properties,^1−3^ making them suitable for a range of technological applications. Most notably, epoxies are used as adhesives^4^ and coatings^5^ in the automotive industry, as well as matrix materials in various composites.^6^

They are typically prepared from an initially liquid mixture of epoxy prepolymer and so-called hardener molecules, which chemically react to form a covalently cross-linked network structure. This reaction is referred to as curing. If a suitable combination of hardener, epoxy, and reaction conditions is chosen, the curing reaction will proceed until the continuously expanding network of covalent bonds spans the entire sample. At this point, the initially liquid mixture is converted into a solid gel,^7^ and the structure of the material is irreversibly set.^8^ Nonetheless, the curing reaction may proceed further because the network contains some still unreacted functionalities. Ideally, an almost fully cross-linked amorphous material is made, which, upon cooling below the glass transition temperature *T*_g_, resembles a solid glassy material ready to be used in applications.^9^

The thermomechanical properties of the cured epoxy-based materials are directly determined by the chemistry of the monomers and how they combine to form the covalently cross-linked molecular structure.^8,10^ It is therefore not surprising that a rich variety of molecular modeling approaches have been proposed, which aim at a fundamental understanding of the material properties at the molecular scale.^10^ Nevertheless, from the simulation perspective, modeling such a process, which may involve thousands of interacting sites in typical MD systems, is highly challenging. The first attempts involved Monte Carlo techniques based on both kinetic-theory differential equations^11,12^ and off-lattice direct simulations, in which the coordinates of reactive groups were stochastically distributed, with cross-linking proceeding according to the capture-sphere mechanism.^13,14^ Alternative attempts relied on linking together several copies of a single preconstructed dendrimer-like oligomer.^15^

Later studies began to incorporate the ability to simulate cross-linking events using molecular dynamics (MD) with molecular mechanics (MM) based force fields.^10,16−40^ Such approaches have found widespread use and have proven to be an effective tool to explore the behavior of epoxy-resin networks at the molecular level and to derive physical properties such as glass transition temperature, coefficients of thermal expansion, and elastic moduli, among others.^10,18−20,27,30,31,34−38^

Recently, some of these molecular dynamics approaches have been coupled to machine learning techniques to optimize the chemical composition of the epoxy network with respect to a set of desired material properties.^41^

Although the approaches presented above principally aim at an atomistic description of the curing reaction, coarse-grained (CG) models also have been applied to model the curing of epoxy resins at scales much larger than those accessible by MD simulations.^42−44^ Liu et al. conducted cross-linking at the coarse-grained level using dissipative particle dynamics (DPD) simulations, whereas mechanical properties and glass transition temperature were extracted after back mapping the CG beads to an all-atom system.^42^ While the CG DPD model comprises a total of ∼250,000 beads, the back-mapped atomistic systems are much smaller so that only a part of the CG system may be utilized. Other CG approaches may not strictly rely on a back-mapping to an all-atom description; however, this step would always become essential to derive a fully atomistic condition. Unfortunately, no method has been universally accepted to conduct such a back-mapping.^42,45^

Classical MD simulations are, therefore, the method of choice to study epoxy resins even today if a fully atomistic topology is desired. Interestingly, besides the fact that the curing reaction affords the description of a chemical reaction, most atomistic studies rely on nonreactive or fixed-bond force fields. Since nonreactive molecular force fields cannot account for bond breaking and forming, the simulation of the curing reaction usually involves an iteration of three steps (embedded into classical molecular dynamics), the first being a reactive-groups search followed by a topology change and the structural relaxation of the reacted system.^17,33^ The identification of reactive pairs evokes a change in connectivity and in the molecular structure of the system, which in turn requires an update of the molecular mechanics parameters defining the molecular structure after the reaction. This is necessary to continue with the time propagation of the system after (local) structural relaxation.

Although the formulation of these steps is, in principle, universal, details of their implementation may vary significantly. For example, the change of connectivity is typically based on finding predefined binding partners within a cutoff distance. The latter is often increased over time to facilitate further searching of reacting atoms in a progressively less diffusive environment. While typical search radii of 4–10 Å are applied,^27,30−32^ values up to 14 Å have been used to achieve higher curing rates.^29^ Indeed, recently, a very promising new approach has been presented by Konrad et al. that avoids definition of a cutoff radius by streamlining the curing reaction using Morse potentials.^20^ Unfortunately, this approach requires coarse graining of a part of the reacting system and, again, does not allow a fully atomistic treatment of the system of interest.

The challenges involved in modeling the curing reaction are further aggravated by the polar nature of epoxy resins. As the constituents chemically react during the curing process, the intramolecular charge distribution changes dramatically, whereas the overall system remains electroneutral at all times.^10,21,26,33^ It is well established that the treatment of electrostatic interactions plays a key role in the bonding dynamics of the polymerizing system.^10,16,21,25,26,33^ However, the effects of charges on the resulting topologies and the thermomechanical properties of the system are much less appreciated. The internal distribution of charge should be vital for the understanding of any chemical process involving epoxy resins, including their response to large mechanical stress or aging. It is thus surprising that many computational studies investigating the polymerization reaction of epoxy resins ignore charges altogether (e.g., by using CG models^43^) or show a lack of methodological detail regarding the derivation procedure of partial atomic charges, as noted recently by Demir and Walsh.^16^

The need for correctly updating atomic charges in an atomistic description is well known and was analyzed in more detail by Li and Strachan.^10,25,26^ These authors established a procedure using the DREIDING^46^ force field in conjunction with the electronegativity equalization method (EEM) and the electronegativity-equalization-based charge assignment method (ECA).^47,48^ The problem of deriving accurate and reproducible partial atomic charges for the intermediates appearing during the curing reactions was also addressed by Demir and Walsh,^16^ who presented a new and reproducible approach using the charge equalization method (Q_Eq_).^49^ Both groups resorted to the “activated” forms of epoxy and amine, although the latter does not correspond to any chemical intermediates of the generally accepted curing reaction mechanism.^50^ Besides determining appropriate partial charges, these approaches also maintain charge neutrality of the system. Both EEM and Q_Eq_ are transferable to other systems and have been successfully applied to polymerization reactions,^16,25,28^ but both methods remain computationally demanding because they incorporate on-the-fly charge calculations and continuous updating of the atomic charges during MD simulations.^10^ This can easily become a bottleneck in the case of very large systems, comprising numerous rounds of cross-linking.^10^ Indeed, previous studies utilizing the Q_Eq_ method performed charge updates only once at the beginning for the unreacted system and again only once after the curing reaction was fully terminated. Such a procedure will reduce the computational cost; however, it does not take into account changes of the electrostatic environment during curing.^16^ Indeed, the ECA method developed by Li and Strachan was a first step in the direction of streamlining the generation of partial charges and replaces on-the-fly calculations with a predetermined set of partial charges calibrated for the system of interest.^10,25,26^

Introducing charges into the curing simulation should provide a reliable molecular-scale model of an amorphous epoxy resin, allowing one to relate thermodynamic and mechanical properties to the molecular structure of the resin. However, all these properties depend on the thermodynamic conditions, and their extraction from simulation data often involves approximate procedures. Further difficulty is introduced by the need for extensive sampling and the large system sizes required to capture the complex topology of the macroscopic network.^16,51^ Therefore, obtaining meaningful mesoscopic data comparable to experiments, even with good parametrization, is another demanding task, which, however, needs to be completed to advance the field.

**Figure 1:**
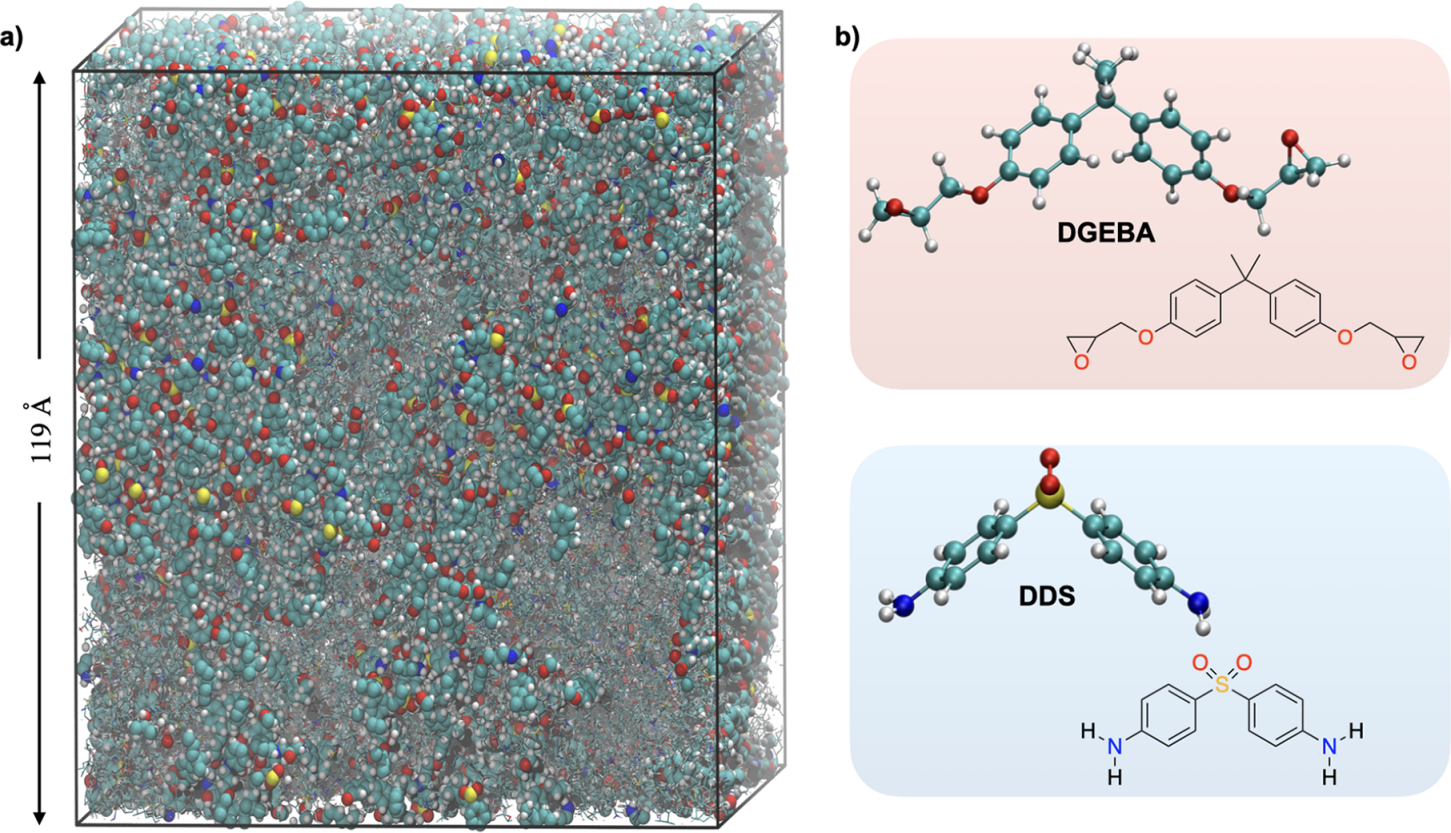
A polymer network emerging in the system during curing. (a) A snapshot from MD simulations of the curing reaction of a DGEBA-DDS epoxy resin of a system with 2000 and 1000 DGEBA and DDS molecules. The box length of the cubic periodic system is indicated by arrows and is approximately 119 Å. The largest cross-linked molecular group is shown as balls and sticks, at the point of percolation. Other groups, which are not part of the largest molecular group, are made transparent. (b) The molecular structures of the precursor DGEBA and the hardener DDS. The block chemistry approach does not necessitate the usage of unchemical preactivated species, and therefore, those structures also represent the actual simulated species.

Our main idea is to transfer the modular strategy adopted by the AMBER force field,^52−54^ which provides consistent partial atomic charges for biopolymers,^55^ to epoxy thermosets. For this purpose, we first develop a block-chemistry approach by parametrizing a clever and minimal set of molecular fragments that can reconstruct the system at every stage of the curing process, including the unreacted liquid precursor. The key advantage of this modular parametrization is the accurate description of the electrostatic interactions evolving during cross-linking without introducing computationally expensive on-the-fly charge recomputations (as required, for example, by EEM and Q_Eq_, as explained above), all the while respecting the overall charge neutrality of the system. The lightweight computational demand of this protocol unlocks the atomistic simulation of the curing process for large systems, in our case up to 127,000 atoms. To showcase our approach, we chose a common epoxy resin system containing DGEBA (diglycidyl ether of bisphenol A) as the resin prepolymer and DDS (4,4′-diaminodiphenyl sulfone) as the amine hardener (Figure 1).

We then expand on the existing bond/react procedures for simulating chemical reactions^23,24,39^ by establishing a three-step protocol that can achieve high curing degrees without relying on ad hoc iterative protocols, preactivated reactive groups, or excessive distance cutoffs. Finally, and importantly, the high degree of physical accuracy of the ensuing model is ascertained by extracting topological, mechanical, and thermal macroscopic properties in statistically rigorous manners. The importance of the electrostatic interactions during curing is also corroborated by systematically comparing topological and thermomechanical properties with those of a system cured in the absence of electrostatic interactions, which, as we will show, exhibit significant deviations from experimental predictions. The results obtained with an adequate treatment of electrostatic interactions during curing, by contrast, are found to result in an excellent match with experimentally available data, showing that the procedures established herein could be utilized in the broader context of quantitative modeling of thermoset polymers.

## Methods

### Development of Partial Atomic Charges

**Scheme 1:**
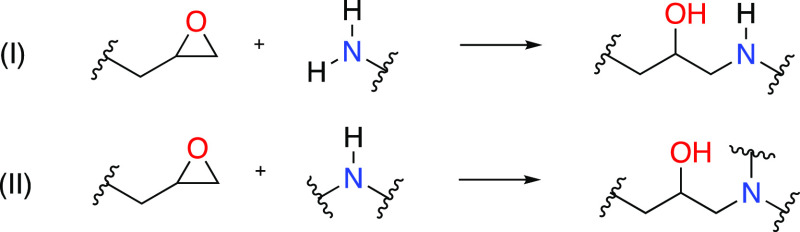
Amine-Based Curing Reactions of Epoxy Resins

During the curing process, a liquid mixture of epoxy and hardener monomers reacts chemically to yield the final cross-linked epoxy material. An example of such chemical reactions is shown in Scheme 1 for amine-based hardeners. For typical amine-based hardeners, the curing reaction proceeds via an addition reaction between an epoxide and a primary amine to yield a cross-linked molecule containing a hydroxyl group and a secondary amine. This secondary amine can further react with another epoxy group to give another larger cross-linked molecule containing a tertiary amine and another hydroxyl group. To obtain a sufficiently cross-linked resin, the epoxy prepolymer needs to contain at least two epoxide groups, which can react with at least two different amine moieties of the hardener. A scheme to derive partial atomic charges should therefore reflect at least the uncured monomeric forms containing epoxy and primary amine moieties and cover the intermediates/products that are formed during the curing process. The updated partial charges should then account for the changes in the chemical functionalities and allow the incorporation of the charges into the developing network while maintaining charge neutrality of the whole system.

**Scheme 2:**
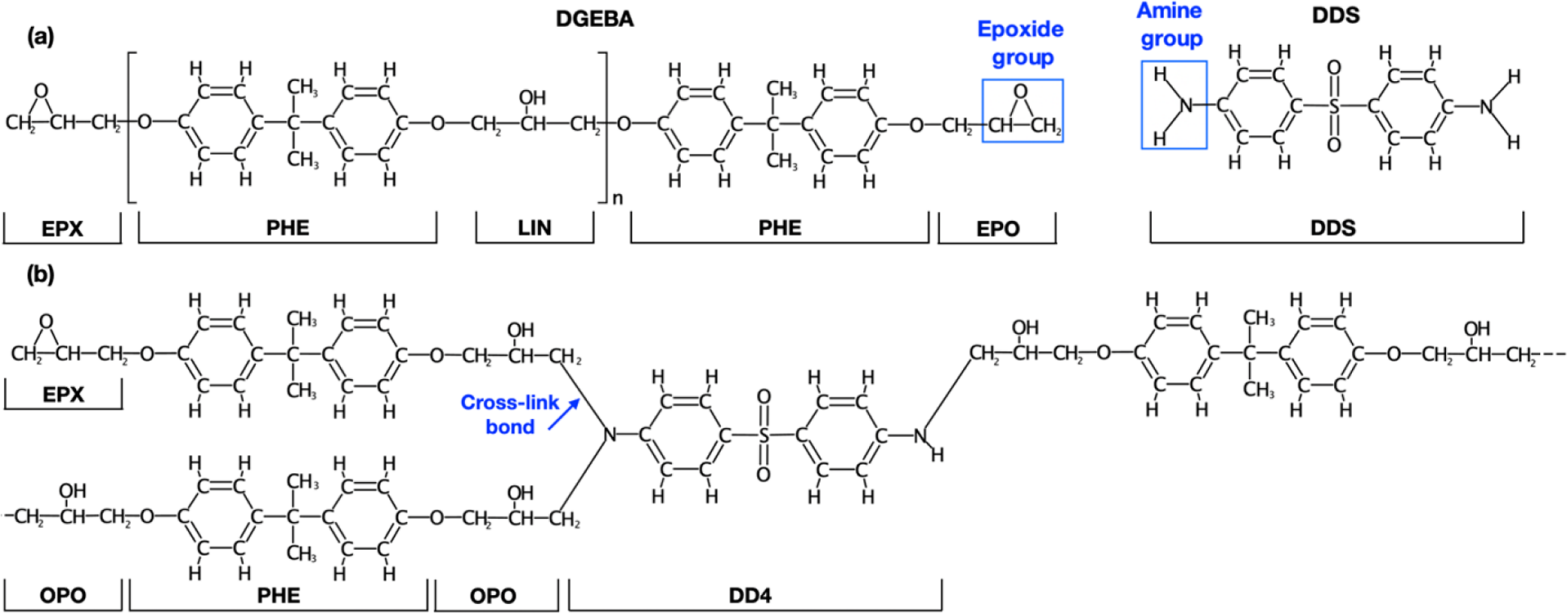
Molecular Constituents Defining the DGEBA/DDS Epoxy Resin and the Definition of the Block Fragments for Charge Calculations Each block fragment has been labeled. (a) A general unreacted DGEBA molecule of length *n*, combining the blocks EPX, PHE, LIN, and EPO and the DDS amine hardener. (b) A possible state of the cured DGEBA-DDS covalent network, which is assembled from the blocks OPO, PHE, OPO, and DD4.

**Scheme 3:**
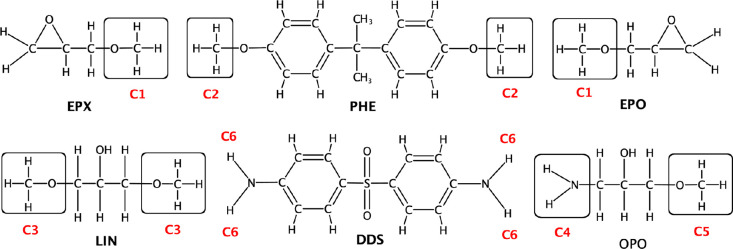
The Block Fragment Defining DGEBA and DDS Epoxy Networks Molecules were split, with capping groups used for charge derivations labeled in red. EPX and EPO are the terminating block units of a DGEBA polymer. For example, a DGEBA molecule with polymerization state *n* = 1 can be recovered as EPX–PHE–LIN–PHE–EPO. The DDS block fragment covers the entire DDS molecule and hence does not require any capping group. C6 is the common charge resulting on each amine hydrogen atom from the charge computation for DDS. OPO is structurally identical to LIN, but one −OCH_3_ capping group was replaced with an amine (C4) to better reflect the postreaction chemical environment in which OPO is linked to DDS by replacing any of the amine hydrogen atoms (C6)

The goal of our charge derivation procedure is to obtain a set of charges that is able to describe any polymerization state *n* of DGEBA (Scheme 2a), the amine hardener, as well as any state of curing (Scheme 2b). The polymeric character of the system suggested a fragment-based approach in which the DGEBA and DDS molecules were broken down into pieces (“blocks”) equipped with capping groups at the extremities (Scheme 3). Charges for each capped block fragment were derived by imposing two simultaneous conditions: both the block itself and a DGEBA molecule of any length, resulting from linking the blocks together and removing the capping groups, had to be charge neutral. For this purpose, appropriate intermolecular charge constraints (C1–C6) were applied during charge derivation.

More specifically, partial atomic charges were derived following the RESP methodology,^56^ as implemented by the RED.III.5 tools^57^ using geometry optimized molecular structures and ESPs obtained with Gaussian 16^58^ at the HF/6-31G(d)^59−62^ level of theory.^52^ The aromatic ether groups of DGEBA were modeled by −CH3 capping groups on the PHE fragment together with −OCH3 capping groups on the terminating EPX/EPO block fragments to account for the chemical environment occurring after those blocks are joined together. We note that EPX and EPO are of course chemically equivalent; EPX simply represents a terminal “starting” block (connecting via the “tail” carbon atom to the next residue) and EPO a terminal “ending” block (connecting via the “head” carbon atom to the previous residue). The DDS molecule was not further split into fragments and was used as a single unit during charge derivation.

**Scheme 4:**
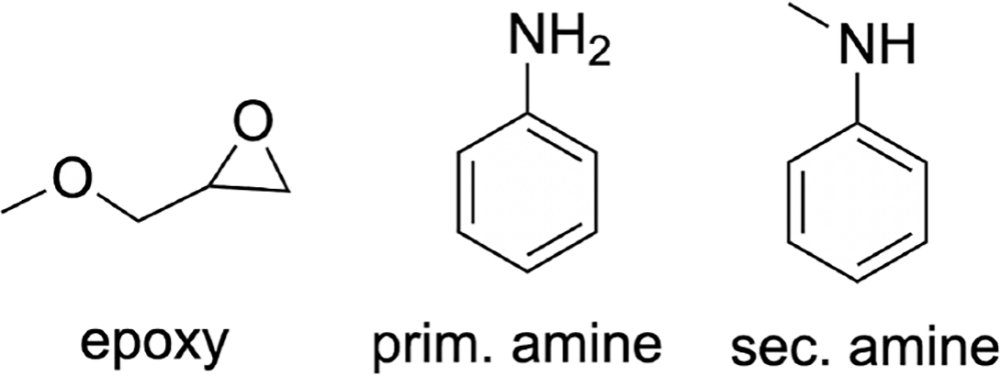
Model Molecules Used in the Characterization of the Primary and Secondary Curing Reaction

**Scheme 5:**
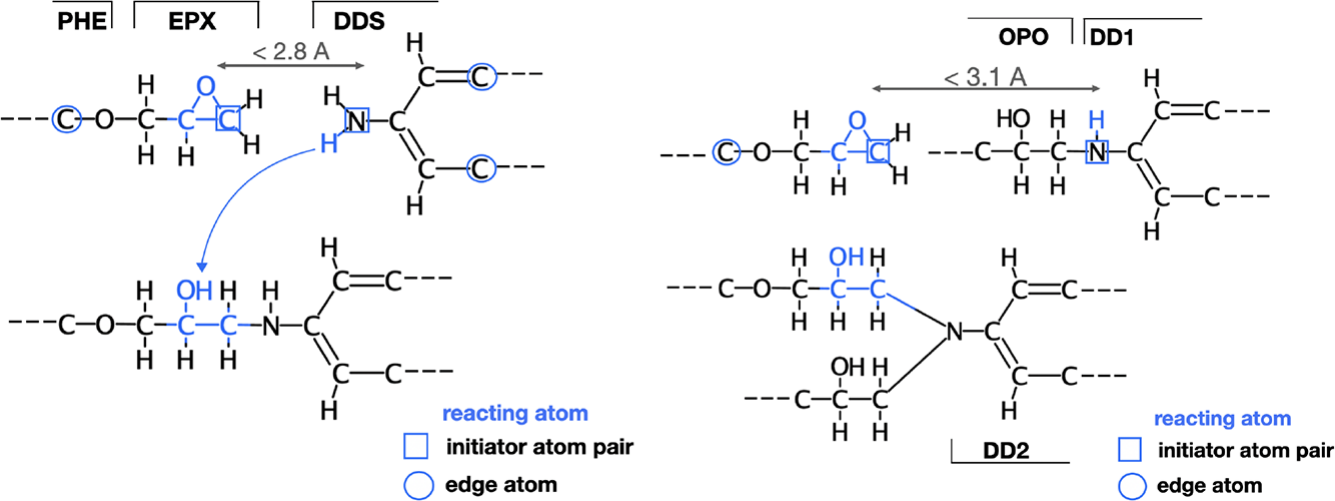
Reaction Site Templates for the First (Left) and the Second (Right) Curing Reaction Situations before (top) and after (bottom) the reaction are presented. “Reacting”, “initiator”, and “edge” atoms are indicated.

One additional block (OPO) is necessary to describe the covalent network during and after the curing reaction (Scheme 3). OPO, the terminal fragment of a DGEBA molecule resulting from a cross-linking event, required charge constraints compatible with the overall charge neutrality of the unreacted system. With this definition of the OPO and DDS fragments, we can describe all six possible curing states of DDS obtained by combining either a primary or secondary amine on one side of the hardener with another primary or secondary amine on the other side. Thanks to the combination of inter- and intramolecular charge constraints (see Figure S1), this drastically simplifies the number of necessary fragments and allows the usage of the reaction schemes depicted in Schemes 4 and 5 for all curing states (details on this procedure follow in the next sections). The final partial atomic charges for all blocks fragments are reported in Scheme S1. The AMBER force field library files of our blocks and all the LAMMPS scripts necessary to reproduce our simulations are publicly available on Zenodo.^63^ With these block fragments, our modular charge scheme can be applied to any cured or uncured DGEBA-DDS network, including any polymerization state *n* of DGEBA.

Notably, the block chemistry principle can also be easily adapted to other epoxy/amine systems at the minimum cost of two charge computations: one for the new hardener and one for the short OPO block linking the latter to the epoxy system. To demonstrate this point, we provide in the repository library files two additional amine hardeners: dicyandiamide (DICY), a nonaromatic tetrafunctional agent, and isophthalate tetramine (IPTA), an aromatic hexafunctional one. This showcases the transferability of the block chemistry approach to other functionalities.

### Building and Equilibrating the Liquid State

Each simulated system consisted of a cubic cell filled with a stoichiometric mixture of 2000 DGEBA and 1000 DDS molecules, for a total of 127,000 atoms. To investigate finite-size effects, we additionally constructed a set of smaller cells comprising 100 DGEBA and 50 DDS molecules (6350 atoms) and 500 DGEBA and 250 DDS molecules (31,750 atoms), respectively. The amorphous starting structures were assembled with Packmol^64^ at a density of 1 g/cm^3^. The smallest interatomic distance between two atoms belonging to different molecules was constrained to be greater than 2.5 Å to avoid occasional ring spearing (see Figure S2 for an example). The initial box length was close to 115 Å for the large systems, 73 Å for the intermediate systems, and 43 Å for the smaller cells. The AMBERTOOLS17 package^65^ was used to output the topology and coordinate files for the five systems. Translation into a LAMMPS (Dec. 2018, stable) data file (available on Zenodo^63^) was then performed by an in-house conversion script. Inter- and intramolecular interactions were described by force field terms and parameters from the original GAFF force field including harmonic functions to describe bond stretching and angle bending. Torsional terms were converted to the simpler harmonic form offered in LAMMPS to speed up the calculations. Lennard–Jones cross-parameters involving different atom types were obtained using the Lorentz/Berthelot mixing rules. Atomic charges were derived as described in the previous sections. All parameters can be found in the LAMMPS data files in our Zenodo repository.^63^

After minimization of the initial geometry using a combination of steepest descent and conjugate gradient, each cell was heated for 20 ps in the *NVT* ensemble, raising the temperature to 503 K, and then equilibrated in the *NPT* ensemble for 2 ns, allowing the density to reach a steady state (see Figure S7 and Zenodo^63^ for input scripts). A Nosè–Hoover thermostat and isotropic barostat at 1 atm with a 100 fs relaxation time, periodic boundary conditions, and a 1 fs integration time step were employed throughout. For each investigated system, five replicas were constructed by repeatedly starting the procedure from scratch.

### The Curing Reaction

To obtain a better understanding of the curing reactions that can take place in the DGEBA/DDS system, we first investigated possible curing reactions occurring in the studied thermoset in the gas phase at the DLPNO-CCSD(T)^66^/def2-QZVPP^67,68^//wB97xD^69^/def2-TZVP^67^ level of theory using small molecular models (Scheme 4). The coupled cluster calculations were conducted with Orca.^70^ For further details on these calculations, please refer to Section S2 of the Supporting Information. All optimized structures (Figure S3) are available on Zenodo.^63^

**Figure 2:**
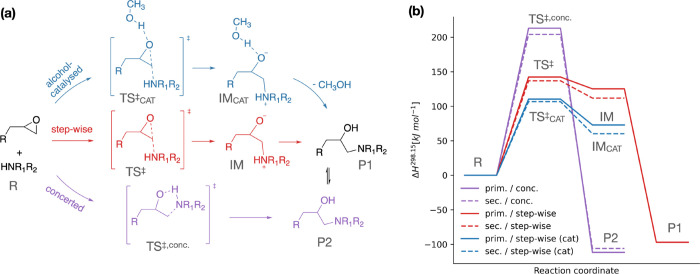
Investigation of the curing reaction involving primary and secondary aromatic amines by quantum mechanical calculations at the DLPNO-CCSD(T)/def2-QZVPP//wB97-xD/def2-TZVP level of theory. The different reaction mechanisms and the computed relative enthalpies are shown in panels a and b, respectively.

The addition of an amine to the epoxide group is generally initiated by a nucleophilic attack of the amine nitrogen at the terminal carbon atom of the epoxy ring.^50^ Although multiple chemical reaction mechanisms have been proposed,^71−74^ Ehlers et al. reported that a stepwise rather than a concerted reaction pathway is favored in the case of aliphatic amines (cf. Figure 2).^50^ Because of the structure of the rate-determining transition state, such pathways have also been termed “cyclic” (i.e., concerted) or “acyclic” (i.e., stepwise). It has been shown that the reaction is catalyzed by hydrogen bond donors either via (unreacted) primary and secondary amines or via the alcohol groups forming in the mixture during the course of reaction.^50,74^ The latter showed the largest catalytic effect.^50^ Here, we investigate the reaction kinetics for model systems involving primary and secondary aromatic amines (Figure 2) taking into account the most prominent stepwise, concerted, and stepwise/alcohol-catalyzed mechanistic pathways.

The obtained gas-phase energetic barriers (Figure 2 and Table S1) for the different investigated reaction mechanisms are in line with the previous findings for aliphatic amines. The concerted pathway is very unlikely due to a large energetic barrier (above 200 kJ/mol), and the formation of the zwitterionic intermediate is the rate-determining step in the stepwise pathway (∼136.9 and 142.3 kJ/mol for primary and secondary amines, respectively). The subsequent proton transfer is barrier-less in the gas phase. The overall reaction is highly exothermic, and the large reverse barrier of the concerted mechanism excludes any back reactions following this pathway (>300 kJ/mol). The zwitterionic intermediate is also not thermodynamically favored in contrast to previous reports investigating BFDGE (bisphenol F-diglycidyl-ehter) and DETDA (3,5-diethyltoluene-2,4-diamine) epoxy resins,^20^ suggesting that the reaction surface can be strongly influenced by the choice of the monomer composition.

The presence of hydroxyl groups can, however, strongly facilitate the stepwise pathway, here exemplified by the alcohol-catalyzed reaction. The formation of hydrogen bonding interactions reduces the gas-phase barrier to 110.3 and 106.6 kJ/mol for the reaction with primary and secondary amines, respectively. Nevertheless, experimentally determined barriers for the reaction of DGEBA with aromatic amines were indeed between 51^75^ and 74 kJ/mol,^76^ which are still significantly lower than our best estimates in the gas phase.

**Figure 3:**
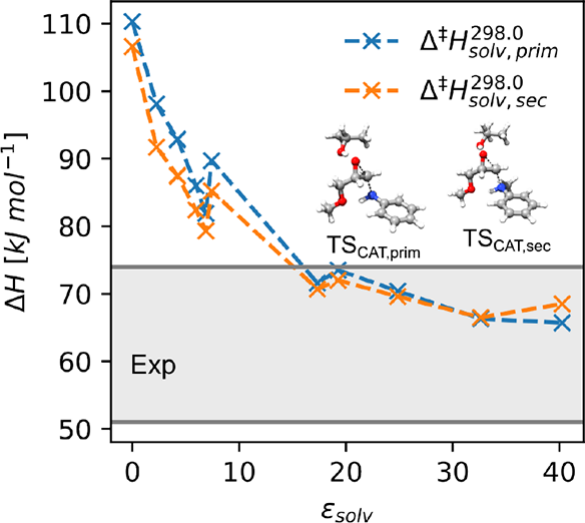
Estimation of environment effects on the barriers for the first elementary step of the alcohol catalyzed curing reaction with primary and secondary amines. The enthalpic barrier Δ^‡^*H* for the alcohol catalyzed stepwise reaction is plotted as a function of the dielectric constant ε_solv_.

We hypothesize that the difference between experimental measurements and our gas phase predictions arises due to the dielectric properties of the environment in which the reaction is taking place, which, unfortunately, is not known. We therefore computed the effect of different dielectric environments on the energetic barrier with the SMD^77^ model at the wB97XD/def2TZVP level of theory (Figure 3 and Table S2), which much better reflects the situation in the initial liquid state. From this data, it is evident that the environment’s effect can be estimated to lie between −25 and −45 kJ/mol, effectively lowering the estimated barrier to 83–63 kJ/mol, in perfect agreement with the experimental data.

Nevertheless, the primary aim of our study is not to produce highly accurate reaction barriers or the derivation of accurate reactive force fields from this type of calculations. It has been shown many times that the curing reaction can be accurately modeled via alchemical approaches neglecting or streamlining the reaction barriers^16,20,22,24,40^ simply because equilibrium properties depend solely on the relative free energy gain and not on the barrier. Furthermore, it has been recognized that the energetic barriers of the individual reactions are very prohibitive for time scales observable with MD simulations and typically need to be artificially smoothed out or boosted to allow any reaction to take place in accessible time frames.^18,20,24^ The inclusion of barriers would be necessary if the competing reactions (i.e., between primary and secondary amines) occurred on completely different time scales, which would indeed affect the properties of the cured material,^22^ e.g., by producing long-lasting meta-stable networks. However, if both reactions follow identical kinetics, this is not a problem. This fact is deeply rooted in statistical physics and can be demonstrated by comparison with experimental data,^22^ as conducted in the following sections.

Thankfully, in our case, the differences between the barriers for primary and secondary amines for the rate-determining steps are very small (5.4 and 3.6 kJ/mol for the stepwise and stepwise/alcohol-catalyzed pathways, respectively). Such energetic differences are even smaller or close to the typical errors expected for computed energy barriers at this level of theory.^78^ Thus, we can safely neglect the small differences of the kinetic constants for primary and secondary amines and assume identical reactivity for both types of reactions. We will furthermore assume that the curing reaction is not reversible at the timescales of our MD simulations and follow previous strategies in modeling the reaction with fixed-bond force fields.^23,24,39^ In this type of calculations, the reaction partners are identified via a distance cutoff between the reactants. Whereas previous studies typically utilized “activated″ forms of amine and epoxy, we will start our simulations from a more realistic mixture of unreacted DGEBA and DDS monomers. We will, however, also streamline the reaction by neglecting the zwitterionic intermediates, which are assumed to be very short-lived species,^50^ and directly convert the topology into the thermodynamically stable cured form. The details of this procedure as well as the pre- and postreaction topologies are summarized in the following.

Our pre- and postreaction templates are shown in Scheme 5 and are available on Zenodo.^63^ The first reaction was allowed to occur whenever the N atom of a primary amine found itself at a distance less than 2.8 Å from a terminal C atom of an epoxide group. Analogously, the search cutoff for the second reaction was slightly increased to 3.1 Å to account for the fact that the reaction between a secondary amine and an epoxide occurred in an already cross-linked, so more crowded, environment.^16,33^ It should be noted that the two search radii were kept constant at all degrees of cure. On the one hand, this was to avoid high-strain configurations involving cross-link bonds between chains too far apart; on the other hand, short cutoffs also prevent the occurrence of ring spearing (Figure S2).

This procedure was applied to both a system with fully parametrized block chemistry fragments and one leaving out all Coulomb interactions (see SI, Section 8). This will allow us to assess the impact of partial atomic charges on the physical properties of the cured materials.

### The Cross-Linking Simulation Protocol

The actual curing simulation was conducted in LAMMPS^79^ and consisted of three main steps in the outer loop: a curing step, annealing of the system, and equilibration (Figure 2). These steps were iterated until 95% cross-linking was exceeded. The LAMMPS scripts developed for the implementation of this protocol are available on Zenodo.^63^

Curing cycles were performed using fix bond/react,^23^ which executed its own inner loop consisting of three processes (Figure 2): reactive-group search, topology change, and structural relaxation. Specifically, bond/react effected accurate topology updates from the pre- to the postreacted configurations (Scheme 5) triggered by a distance cutoff between reacting atoms. These changes were part of an ordinary MD integration step and were followed by a relaxation procedure local to the reaction site. Template files had to be supplied describing the topology of any desired pre- and postreaction sites, which were identified in the simulation box by means of a superposition algorithm. Transition from the pre- to the postreacted configuration involved updating all topological parameters, including partial atomic charges (when present), Lennard–Jones parameters, as well as bond, angle, and dihedral coefficients. After a cross-linking event, all reacting atoms involved were stabilized for 60 fs by restraining their displacements to a maximum of 0.5 Å per step.

To increase the mobility of molecules as much as possible while still retaining a temperature close to experimental conditions, the curing temperature was set to 503 K (230 °C).^80^ Furthermore, to allow the volume of the system to dynamically shrink in response to cross-linking, the reaction was performed in the *NPT* ensemble at isotropic atmospheric pressure. Other than the very first curing cycle, which was set to a total duration of 4 ns to exploit the initial high mobility of the reactants, every following curing cycle in the loop was 2 ns long. Furthermore, over the course of curing, both connectivity and coordinate data were dumped every 1 ps for later analysis and characterization of the system. The 1 fs time step of the inner loop coincided with the time step of the MD integrator.

The curing extent is defined as the total number of reactions observed divided by the maximal possible number of reactions. This means that the reaction or curing extent is bracketed by the unreacted liquid (0%) and the hypothetical fully cross-linked system for stoichiometric amounts of amine and epoxy functionalities (100%). If a curing cycle was allowed to proceed until no further reaction took place, curing intensities of about 80% would be achieved, with most of the cross-linking occurring within 4 ns (Figure S4). Even though the probability for the curing reactions increases with crowding, the small cutoffs make sure that only a few reactions take place within a curing step and that the formation of secondary amines precedes the formation of tertiary amines (Figure S5).

**Figure 4:**
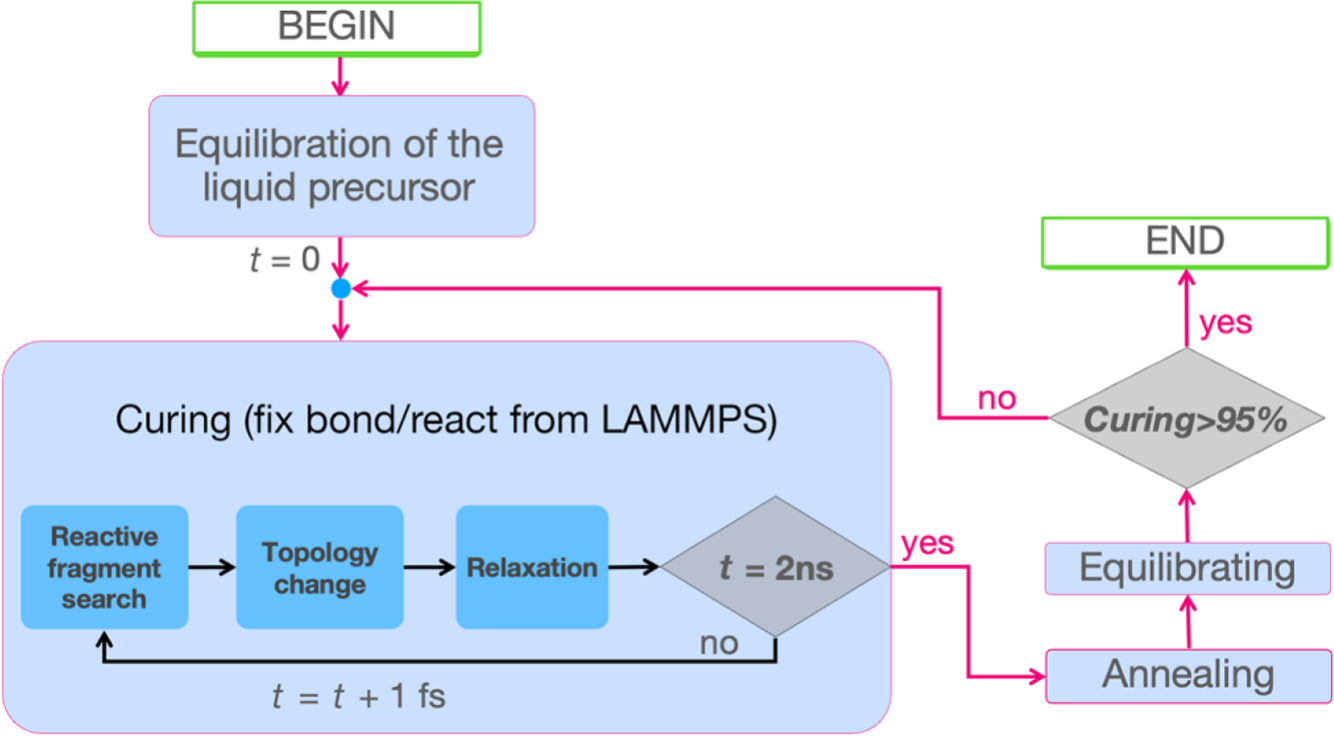
Flowchart representing the MD curing procedure preceded by the initial equilibration of the liquid mixture. Curing periods were followed by annealing and equilibration phases until the target curing extent of 95% was reached. At each time step within a curing period, fix bond/react identified relevant reaction sites, modified their topology as necessary, and finally applied relaxation.

To achieve high curing rates without introducing artificially large distance cutoffs, we implemented a mixing procedure during which no cross-linking reaction was allowed. This involved an annealing run (Figure 4) followed by an equilibration run (see Figure S6 for an example iterative run). Specifically, for curing degrees below 90%, after every curing cycle, the temperature was increased to 800 K over 30 ps in the *NVT* ensemble, kept constant in the *NPT* ensemble for 500 ps, and then reduced again to 503 K. When exceeding 90% curing, the annealing time in the *NPT* ensemble was progressively increased up to 2.5 ns in 500 ps increments. Each annealing round was followed by an equilibration step involving 100 ps of *NPT* equilibration at 503 K to let the density stabilize before the next stage of cross-linking (see Figure S8 for a discussion of the impact of doubling this equilibration period on the physical properties of the material).

Following the equilibration, the curing step was repeated until 95% conversion was achieved. This level of cross-linking well surpasses the results of previous coarse-grained models simulating much larger systems,^42^ with reasonable computational costs. The latter was benchmarked against an ordinary *NPT* run showing that the overhead is not more than 65% (see Section 5 and Table S3 of the SI, where we also provide technical details of the underlying hardware).

## Results

### Measurement of the Gel Point

As the curing protocol proceeds, the liquid turns into a gel. The point of gelation for a cross-linking system is defined as the extent of reaction corresponding to incipient chemical network formation.^7^ According to the classical Flory–Stockmayer^81,82^ theory of polymerization, gelation coincides with the emergence of a macroscopic molecule, the “gel”, which extends throughout the volume of the mixture and whose mass is far greater than that of single monomers.^83^ The theory describes gelation solely in terms of the functionalities of the reactants, predicting percolation exactly at 58% for the present case of a tetrafunctional hardener (DDS) and a bifunctional resin (DGEBA).

Traditionally, gelation has been measured from MD data only indirectly by looking for significant changes in some physical or structural properties related to the inception of network formation. This usually includes the largest mass buildup,^22^ the onset of intramolecular polymerization reactions,^84^ or the reduced molecular weight (RMW).^12,85^ Recently, we have investigated the limitations of these methods and introduced a rigorous algorithmic procedure to exactly measure the gel point in MD simulations.^51^ Our strategy measures the gel point as a percolation transition. In the limit of a macroscopic system, in which the size of the network is by far greater than the size of any single monomer, the periodic percolation threshold approaches the actual percolation point of the system. A portrait of one of our cross-linking epoxy models at the point of periodic percolation is given in Figure 1.

**Figure 5:**
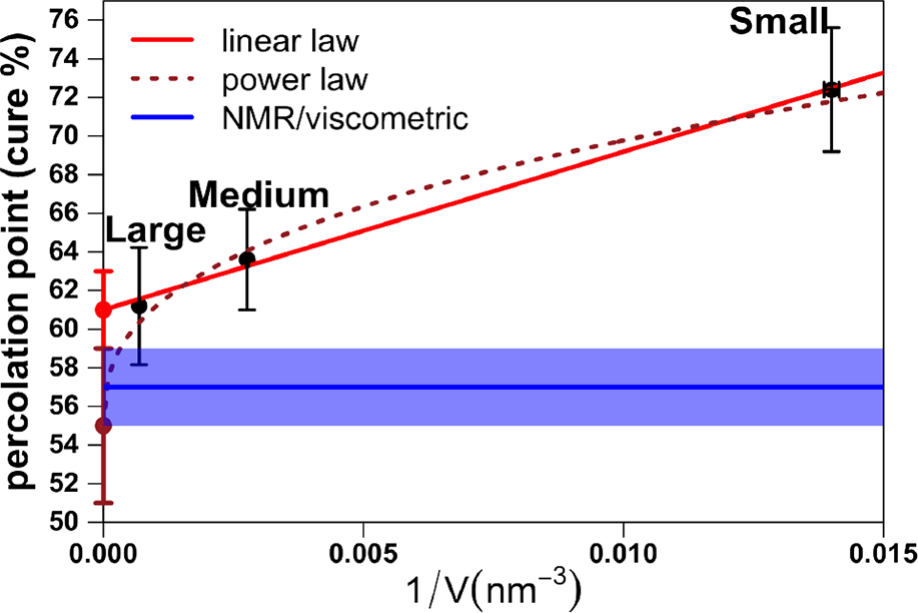
Comparison of gel point results with experimental NMR/viscometric measurements,^86^ both expressed as curing percentages. For each system size (large: 127,000 atoms, medium: 31,750 atoms, and small: 6350 atoms), the results were averaged across five independently cured MD samples. Two different approaches were used to extrapolate the percolation point for an infinite (macroscopic) system. Linear fitting (red line) extrapolated such value as *p*_∞_ = 61 ± 2%, whereas fitting the power law (brown dotted curve) *p* – *p*_∞_∼*V*^–1/(3ν)^, expected for a system belonging to the 3D percolation universality class, yielded *p*_∞_ = 55 ± 4% The experimental range 57 ± 2% was shaded in blue.

As expected, the results of our simulation depend strongly on the size of the system; see Figure 5. To extract the value of the percolation point for a macroscopic system, we extrapolated the results in the limit 1/*V* = 0 according to two different models. First, motivated by the linear alignment of our MD results, we used simple linear regression and estimated the percolation point of an infinite system as *p*_∞_ = 61 ± 2%. The second approach is based on the universality of critical phenomena. Quite generally, the transition in finite systems is expected to occur when the correlation length ξ∼|*p* – *p*_∞_|^–ν^ is of the order of the linear size of the system *V*^1/3^. For percolation in three-dimensional systems, the critical exponent is υ ≈ 0.88,^87^ which gives the curing percentage *p* – *p*_∞_∼*V*^–1/(3ν)^ ≈ *V*^–0.38^. This fitting is plotted with a brown dotted curve in Figure 5, and it gives the estimate *p*_∞_ = 55 ± 4%. Both of our predictions for the macroscopic percolation point compare very well with the experimental result of 57 ± 2% obtained by viscometric measurements coupled to monitoring of reaction extents by NMR.^86^ They are also in agreement with the percolation point at 58% found for the material cured without partial atomic charges, further suggesting that indeed the functionality of the reactants is solely responsible for gelation. However, the results of our simulations do not allow us to clearly distinguish between the two proposed power laws even though our largest system exceeds typical MD setups. It thus remains to be clarified if the observed transition indeed belongs to the 3D percolation universality class.

### Measurement of the Glass Transition Temperature

As the polymer blend undergoes cooling or even compression, the molten fluid changes into a brittle glassy material. This is accompanied by an important change in the heat capacity of the system and its elastic constants and thermal compressibility, among other properties. The transition is associated with a characteristic temperature, denoted here as *T*_g_.

**Figure 6:**
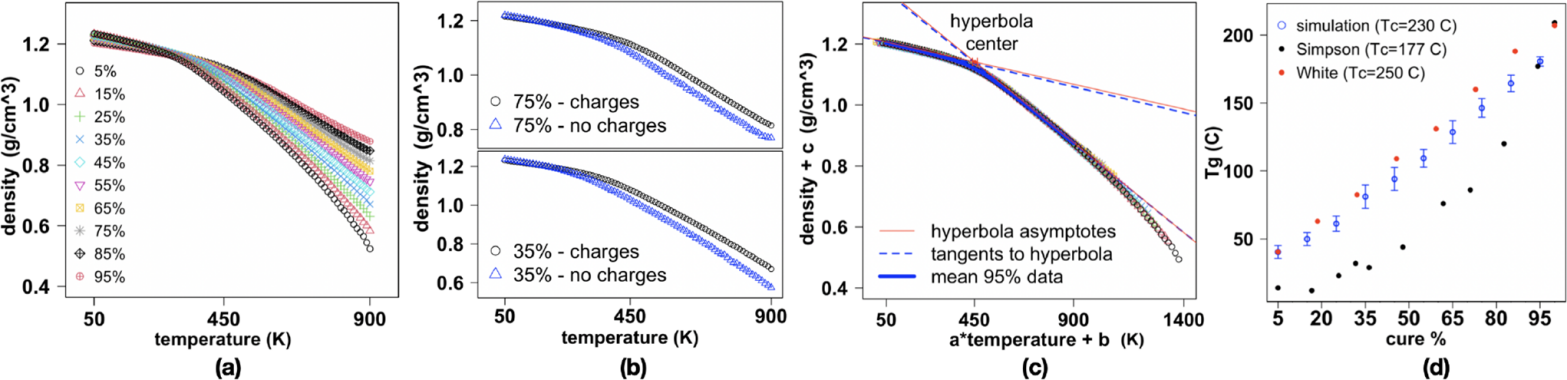
Characterization of the glass transition temperature. (a) Plots of density as a function of temperature, at each investigated curing percentage, each averaged across the five systems. (b) Example plots of density as a function of temperature for charged and uncharged systems. (c) Master curve obtained for all simulated systems. The excellent agreement between analytic tangents and asymptotes testifies to the quality of the fit. (d) *T*_g_ values as a function of cure percentage, averaged over all five systems, with error bars as given in Table S4. The two sets of solid dots represent experimental data from White et al.^80^ and Simpson and Bidstrup,^89^ which are in line with earlier works^90^ and were originally reported without error bars.

To study the glass transition temperature, restart files were generated during the curing simulation after every 10% increment in the degree of curing, starting from 5% and ending at 95%. Each of these partially cross-linked systems was heated up to 900 K at a rate of 10 K/ps and then slowly cooled down to 50 K in 10 K decrements applied at the same rate. After each temperature drop, each system was equilibrated in the *NPT* ensemble for 100 ps at a pressure of 1 atm using the Nosè–Hoover barostat with a relaxation time of 100 fs, allowing the volume and consequently the density to adapt (input scripts provided on Zenodo^63^). Such equilibration period is widely sufficient to obtain a density value in agreement with the long-term mean (see Figure S9 for details). The determination of *T*_g_ (Figure 6) relied on 100 ps production runs, whereas density data were collected every 100 fs.

We find, as expected, that as *T*_g_ is approached from the liquid state, the slope of the density as a function of temperature increases toward the solid phase (Figure 6a). Somewhat surprisingly, we also find that this trend is further enhanced in data sets that explore temperatures far from the transition (low curing, high *T*). Similar trends are observed in the system without charges at 35 and 75% curing (Figure 6b). However, the slopes of the low-temperature tangents are different at the same curing fraction, and the glass transition region is shifted to lower temperatures. Additionally, the density of the material polymerizing without electrostatic interactions is, at temperatures higher than the transition, systematically lower than the one obtained by using charges. It appears that without charges, the system effectively behaves as the one with charges but at smaller curing densities. This is consistent with previous work of Demir and Walsh who also reported lower densities for the systems deprived of Coulomb interactions compared to those of the charged systems, albeit in the liquid phase.^16^

Most notably, we observe that all the block chemistry data can be collapsed onto a master curve (Figure 6c), where the 95% data is used as reference. Each data set was rescaled by both shifting and stretching in the horizontal direction and by shifting over the vertical axis (Table S3). The observed collapse of the density curves ρ(*T*) calculated from the simulations onto one master curve can be related to the universality of glass transition, as expected. As shown in Figure 6c, the transformation depends on three fitted parameters: *a*, *b*, and *c*. Whereas *b* and *c* are used to move the center of each hyperbolic-like curve to the same point, the rescaling of the temperature axis by factor *a* maps the parts for *T* < *T*_g_ and for *T* > *T*_g_ onto a single master curve at the same time. A closer analysis shows that this is only possible when the ratio of the derivatives of ρ(*T*), calculated for the linear part of the curve below and above the glass transition, does not depend (up to numerical errors) on the curing percentage.

To quantify this property, we look at the isobaric thermal expansion coefficient , where the derivatives are calculated at a fixed pressure. In the vicinity of the transition point *T*_g_, this coefficient is expected to follow a power law , where the amplitudes γ_0_^–^ and γ_0_^+^ (valid below and above the transition temperature, respectively) and the transition temperature *T*_g_ are not universal; i.e., they can depend on the curing percentage. The exponent α (the same exponent describing the behavior of isobaric specific heat) and the ratio of amplitudes γ_0_^–^/γ_0_^+^ are universal and should not depend on the curing percentage. If we assume that α = 0 (without logarithmic corrections) and that the hyperbolic shape of the curves around *T*_g_ is caused by the smooth crossover between glass and liquid phases, due to the nonzero speed at which the temperature was changed in the simulation,^88^ the proposed power law seems to agree with our numerical results. The jump of the derivative of the curve ρ(*T*) at *T* = *T*_g_ for an idealized, infinitely slow process can be estimated by fitting lines to linear parts of the curves obtained from the simulation for *T* < *T*_g_ and for *T* > *T*_g_. This way, we verified that, up to numerical errors, the ratio of amplitudes is the same for all curing percentages, and we estimated it to be γ_0_^–^/γ_0_^+^ = 0.260 ± 0.004. This universal behavior, observed to the best of our knowledge for the first time in MD simulations, independently shows that our block chemistry methodology is suitable for modeling epoxy networks.

In ideal conditions, the change of the density with temperature follows a hyperbolic function. Therefore, the traditional way of extracting *T*_g_ values from annealing trajectories consists of plotting the evolution of density as a function of temperature, fitting one line to the low- and another line to the high-temperature tails of each data set independently, and then taking *T*_g_ to be the temperature at which these two empirical tangents intersect.^91,92^ However, the result of this procedure depends strongly on the number of data points included in the fitting of the two tails. An alternative analysis was proposed by Patrone et al.,^93^ in which the empirical tangents to the low- and high-temperature branches are replaced by analytic tangents of the hyperbola fitted to the entire data set. Because the fit is unique, the ambiguity is removed (see Figure S10). In the ideal case when the asymptotic behavior is well captured by the simulations, both procedures should give identical *T*_g_. However, because the transition point shifts with the curing intensity, the asymptotic behavior may not have been achieved in the window of realistic temperatures. For example, data sets obtained for small curing degrees have a very short region in the liquid state, and obtaining asymptotic behavior is not possible in this case. One further limitation of both methods is that the density data sets are not showing a hyperbolic trend at temperatures away from *T*_g_. Thus, addressing these data sets using hyperbolas that rely on the extremities of the studied temperature window may lead to wrong estimation of the glass transition (Figure S10).

A simple solution to these issues that permits a reliable measurement of *T*_g_ is fitting a master curve. All sets combined reconstruct the full dependence of the density as a function of temperature. The hyperbola can then be fitted only close to the transition point (Figure 6c), at which the analytic tangents and the hyperbola asymptotes basically coincide. *T*_g_ can then be extracted by applying the inverse rescaling. The inverse transformation and the scaling parameters, as well as their uncertainties, are reported in Table S4. Aggregate results for all five systems are shown in Figure 6d. This panel also offers a comparison of our simulation measurement (blue dots) with experimental data obtained using differential scanning calorimetry^80^ (DSC) on systems cured at 250 °C (red dots in Figure 6d) and rheological techniques^89^ obtained from systems cured at a temperature of 177 °C (black dots). The relative vertical shift of the experimental measurements shows that a greater cross-linking temperature leads to a higher *T*_g_ value at each curing extent.

Our results for *T*_g_ show excellent agreement with experiments (Figure 6d). The transition temperature monotonically increases with curing percentage. Furthermore, as our systems are cured at 230 °C, the transition temperatures are slightly but systematically below the DSC measurement at 250 °C and well above the rheology measurement at 177 °C. By contrast, the *T*_g_ values measured in the systems cured in the absence of partial atomic charges showed significant deviations. Specifically, at 35% curing, *T*_g_ drops from 78 ± 11 °C in the system with charges to 28 ± 9 °C in the one without charges. Similarly, at 75% curing, the decrease is from 145 ± 11 to 94 ± 10 °C. This could imply a different slope in the *T*_g_ than observed with charges but also that the system without charges behaves as the one with charges with densities shifted to lower values. Furthermore, the agreement with the experiment is far from the excellent one achieved by our block chemistry approach at all investigated degrees of cross-linking. This analysis confirms that, with sufficient appropriate analysis of simulation data as proposed by our scaling procedure, reliable results for the glass transition temperature can be obtained from simulations.

### Measurement of Young’s Modulus

We finally determined the Young’s modulus of our system by performing a tensile stress simulation. To make sure that the system was not trapped in a metastable equilibrium, which is known to affect the mechanical properties of MD polymeric networks,^24^ we subjected each cured sample to a postcuring annealing procedure consisting of 10 ns at a temperature of 1000 K (see Zenodo^63^ for input scripts), after which the temperature was quickly lowered to 300 K. Each system was then allowed to equilibrate in the *NPT* ensemble at 298 K using isotropic pressure coupling until its density reached steady state (see Table S5). To rule out the possibility of insufficient annealing time, we also extended the postcuring annealing period up to 20 ns but without observing significant changes in the mechanical response of the system. After completing equilibration at room temperature, each cross-linked sample underwent a uniaxial tensile test along the *x*, *y*, and *z* axes at an engineering strain rate of 2·10^–8^ s^–1^ (input available at Zenodo^63^). This strain rate was chosen as a compromise between the computational efficiency of sampling and straining the system as slow as possible. This choice is motivated by previous studies^10^ where it was shown that the effect of the strain rate on elastic properties is small for sufficiently large systems as ours and negligible compared to the effect of the choice of the force field.

**Figure 7:**
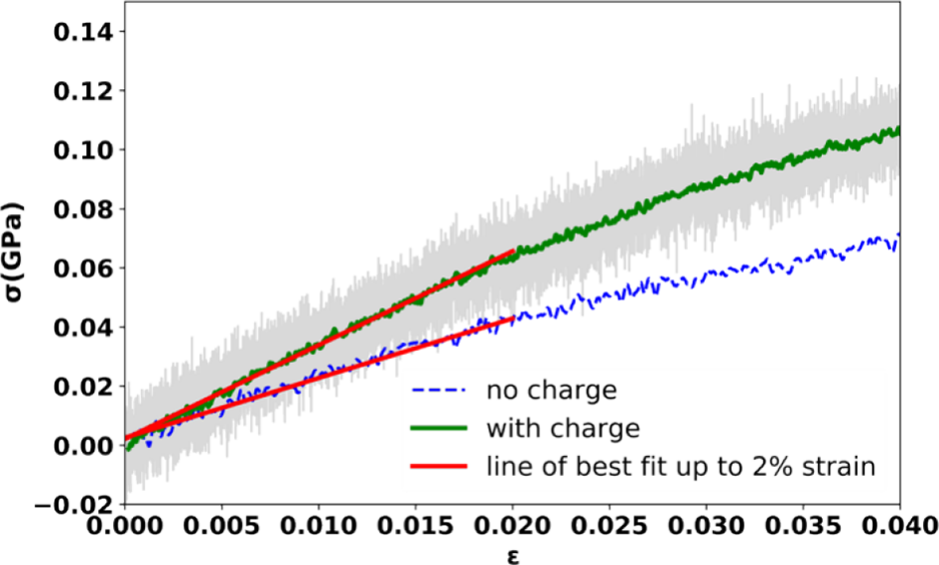
Stress (σ)–strain (ε) plot resulting from averaging tensile test data for all five MD samples (green) and a single MD sample cured with no charges (dashed blue) subjected to deformation. The line of best fit is determined using data only up to 0.02 strain because the plot clearly bends beyond that value. The shaded area surrounding the plot is given by the pointwise standard deviation of the average stress value.

The stress–strain plot resulting from averaging all 15 tests (five systems times three directions, to ensure sufficient statistical sampling) is shown in Figure 7. The plot suggests that the regime of linear response to deformation ends at 0.02 strain and includes the best-fit line up to that strain value. This is true in both block chemistry generated networks and those constructed with no charges. However, the slope of the system cured without charges is significantly lower, which is associated with a 30% decrease in the Young’s modulus relative to the result obtained with the block chemistry approach accounting for electrostatic interactions.

The Young’s modulus obtained for the block chemistry system was evaluated in the tensile test to be 3.176±0.002 GPa. To verify this result, we also calculate the zero frequency Young’s modulus from the stress tensor fluctuations for all five replicas. Here, the system was allowed to reach 298 K in the *NPT* ensemble similarly to the previous runs, but instead of straining and analyzing the pressure fluctuations, we computed the fluctuations in box vectors to calculate the compliance tensor.^94^ Following this procedure for all five systems, we obtain the average value *Y* = 3.12 ± 0.07. This is within 2% of the value obtained from the tensile stress, confirming a lesser reliance of the applied strain rates on mechanical properties of these systems. Our calculated Young’s moduli, furthermore, compare very well with the experimental flexural modulus of 3.25 GPa and reasonably well with the tensile modulus of about 2.9 GPa (corresponding to a curing temperature of 240 °C) measured by Min et al. (see Table 1 and Figure 8 of ref (95), respectively), again validating our overall approach.

## Discussion and Conclusions

In this work, we developed a method for modeling epoxy systems with high accuracy, which is technically easy to apply because it is fully built upon atomistic molecular dynamics. To enable such a study, we first performed a state-of-the-art QM-to-MM description^66,96^ of the curing reaction that allowed us to discern between different reactivities of primary and secondary amines. These results counteract the confusion present in some earlier studies suggesting the concerted pathway to be the favorable one over the stepwise one,^18^ unlike demonstrated herein and previously for aliphatic amines.^50^ Furthermore, given that the reactivities of primary and secondary amines investigated in our case are similar, their effect on the correct sampling of the curing reaction turned out to be negligible for obtaining a proper physical MM description of the cross-linking process.

To build an MD molecular model for epoxy systems such as DGEBA-DDS, we create the so-called block chemistry approach based on the generalized AMBER force field, GAFF.^52^ The latter is based on a robust block-like fragment scheme for the development of RESP partial atomic charges. Thanks to the careful choice of fragments, capping groups, and charge constraints, the model encompasses DGEBA monomers of any length and can describe both primary and secondary amine cross-linking reactions of the DGEBA-DDS system. Such parametrization solves the problem of treating the electrostatic interactions of both the monomers and all the polymerization states of the network in a unified manner without the need for extensive calculations on-the-fly^10^ or introduction of artificially “preactivated” epoxy and amine groups.^10,16^ Instead, chemically realistic molecules are simulated at all levels of curing including the pure liquid, the gel, and the fully cured glass. Finally, the modularity of the scheme makes it easily transferable to amine hardeners other than the DGEBA-DDS system used here for the demonstration of the method, at the cost of only a few structural optimizations. Our concept of partial charges therefore allows for block chemistry force field developments beyond that of our study and a significantly simplified workflow compared to other strategies combining multiple simulation techniques.^42^

Another advantage of our approach is that after parametrizing the force field, only the standard LAMMPS MD platform is required, with curing input scripts that we made publicly available (Zenodo^63^). Hence, our approach is fully transferable and quick to set up and run. Most notably, it is also very efficient as it attains an almost completely cross-linked system (≥95%) without introducing unphysical interaction ranges in systems that are as large as 100,000 atoms.

The effectiveness of the procedure lays the foundation to a full statistical analysis of the molecular dynamics data. Consequently, we are able to fully characterize the topological, mechanical, and thermodynamic properties of the network during the transitions between different phases of matter. The latter can be tackled now by introducing the full width of methods associated with statistical physics that reliably provide macroscopic materials properties from atomistic data. This includes a systematic analysis of finite size effects, scaling phenomena, and linear response.

The rigorous statistical analysis is the basis for the comparison with experiments, which in the past could be done only sporadically. Here, the results emphasize the accuracy of the block chemistry approach and the curing procedures systematically for all investigated properties. This allows us to clearly show that the careful treatment of atomic charges provides clearly superior predictions compared to systems with no charges. For the gel point, which depends only on the functional form of the reactants, the results with and without are comparable, as expected. However, for the glass transition temperature *T*_g_ and Young’s modulus, the block chemistry approach provides high-quality agreement with experimental data, whereas the absence of charges yields strong deviations.

Our gel point investigation is based on the accurate percolation theory that overlaps with the viscometric experimental data. Finite-size effect analysis shows that systems consisting of more than 100,000 atoms are needed to achieve reliable results. In fact, our largest system with 127,000 atoms is already in perfect agreement with the extrapolated result for the gel point prediction.

The study of glass transition temperatures as a function of curing degree reproduces the experimentally expected monotonic increase with increasing cross-linking. This result emerged from determining the scaling for the density–temperature relation at all curing degrees. The consequent evaluation of *T*_g_ as a function of curing is then robust against deviations from the hyperbolic trend of any density data sets, as well as the finiteness of the temperature window explored in the simulations.

Our final test was the uniaxial deformation, which resulted in an excellent agreement with the experimental flexural and tensile modulus of the DGEBA/DDS thermoset. The overestimation of about 2–9% relative to the reported experiments may be rooted in the unphysically high curing rate necessarily imposed by the short time scales currently amenable to MD investigation and is significantly better than the 30% underestimation of the Young’s modulus observed in the system with no charges.

With these results in hand, we conclude that we made a considerable step toward the goal of producing highly cured networks exhibiting experimental-grade thermomechanical properties. The exposed methodological paradigm to parametrizing, simulating, and characterizing DGEBA epoxy polymer networks paves the way for a broadly consistent approach to the modeling of all amine-based epoxy resins. Nevertheless, open questions such as the influence of admixtures of longer DGEBA prepolymers and incorporation of probabilistic rates for simulations of mixtures of different curing agents are still to be addressed in future work. Although developed at the atomistic level, it may lay the foundations for new and systematic coarse-grained curing models, providing for easy and potentially less ambiguous back mapping. In future works, it will be interesting to coarse grain our atomistic systems with, e.g., the MARTINI scheme,^97−99^ which was recently extended to incorporate reactivity.^100^ The MARTINI scheme incorporates Coulombic interactions, which will be crucial to describe the curing reaction and properties of epoxy resins. Further, it provides efficient back-mapping algorithms, which will allow iterative curing reactions from atomistic to CG and back. We are currently investigating different strategies how such a scheme could be utilized to simulate epoxy resins. Furthermore, it may serve as a platform for the development of QM/MM protocols integrating chemistry-motivated bond breakage in simulations of large mechanical loads. These two problems represent natural extensions of the present piece of work, and we aim at addressing them in the future.

## Supporting Information Available

The partial atomic charges necessary to reconstruct the entire charge scheme, the degree of curing as a function of time when no annealing is applied, and the rolling mean analysis used to determine the equilibration time for density measurement during *T*_g_ annealing; a detailed analysis of the energetics of the elementary reactions involved in the curing reaction between epoxides and aromatic amines; computational overhead induced by the curing procedure; and summary of the impact of electrostatic interactions on curing and the physical properties of the cured thermoset (PDF)

## Notes

The data underlying this study are openly available at Zenodo^63^ with 10.5281/zenodo.5795935. Trajectory files and further information can be provided upon request to the corresponding authors.
