# Supplementary material for: Block Chemistry for Accurate Modeling of Epoxy Resins
Source: J Phys Chem B. 2023 Aug 24;127(35):7648–62. doi: 10.1021/acs.jpcb.3c04724 (PMC10493980; doi:10.1021/acs.jpcb.3c04724)
Supplement: Supplementary file 1 — jp3c04724_si_001.pdf [file jp3c04724_si_001.pdf]

Supporting Information for

Block Chemistry for Accurate Modeling of Epoxy Resins

Mattia Livraghi§, Sampanna Pahi§, Piotr Nowakowski•, David M. Smith•,

Christian R. Wick♠§, Ana-Sunčana. Smith•§**

§Friedrich-Alexander-Universität Erlangen-Nürnberg (FAU), Institute for Theoretical Physics, PULS Group, Interdisciplinary Center for Nanostructured Films (IZNF), Cauerstrasse 3, 91058 Erlangen, Germany

•Group of Computational Life Sciences, Division of Physical Chemistry, Ruđer Bošković Institute, Bijenička cesta 54, 10000 Zagreb, Croatia

♠Friedrich-Alexander-Universität Erlangen-Nürnberg (FAU), Competence Unit for Scientific Computing (CSC), Martensstr. 5a, 91058 Erlangen, Germany

1. PARAMETRISATION 3
2. THE CURING REACTION...6
3. THE CURING PROTOCOL...9
4. EQUILIBRATION OF THE EPOXY NETWORK. 11
5. COMPUTATIONAL EFFICIENCY OF FIX BOND/REACT 13
6. CHARACTERISATION OF THE EPOXY NETWORK 13
7. YOUNG'S MODULUS 15
8. IMPACT OF PARTIAL ATOMIC CHARGES ON CURING AND PHYSICAL PROPERTIES. 16
8.1 PERCOLATION POINT 16
8.2 GLASS TRANSITION TEMPERATURE 16
8.3 ELASTIC MODULUS..16
9. REFERENCES..16

1. Parametrisation

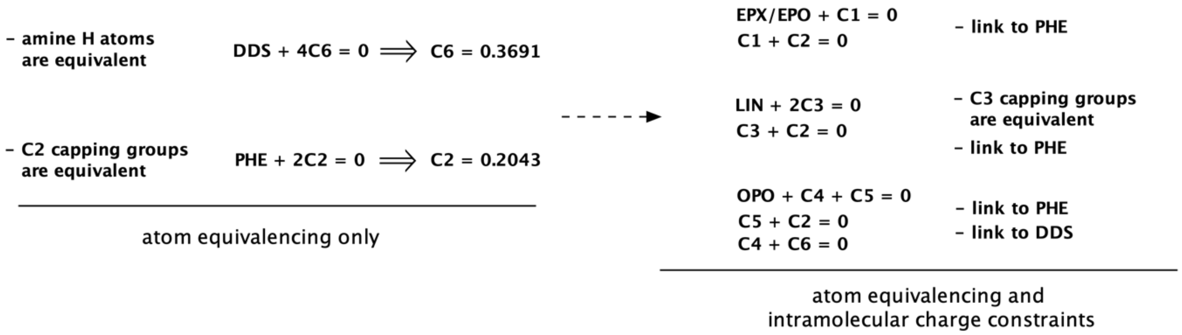

Figure S1. The charge constraints imposed during RESP charge derivation using the RED.III.5 script. Atomic charges were equivalenced as dictated by geometrical symmetry. Intramolecular charge constraints were used to set the total charge of capping groups so that any polymerization state of DGEBA, resulting from appropriate linking of the EPX, PHE, LIN and EPO fragments, was charge neutral. Charge constraints for OPO were set so that any possible cured or partially cured configuration of the DGEBA - DDS network was also charge neutral. For example, the total charge of DGEBA with n=1 is EPX + PHE + LIN + PHE + EPO = - C1 - 2C2 - 2C3 - 2C2 - C1 = C2 - 2C2 + 2C2 – 2C2 + C2 = 0.

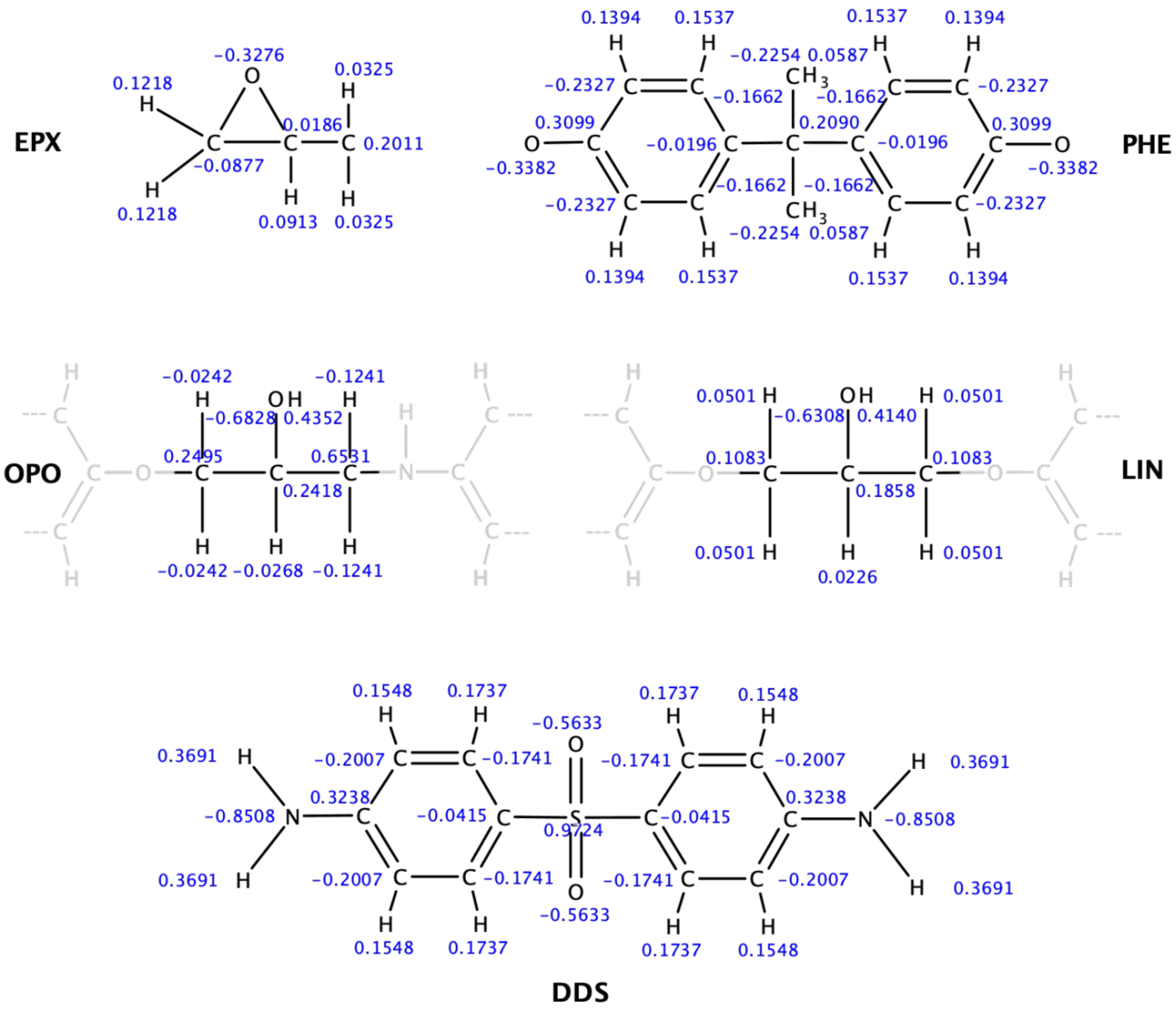

Scheme S1. Partial atomic charges for some of the fragments in our scheme. These charges are sufficient to reconstruct those of the fragments not shown here: EPO has the same charges as EPX; DD1 and DD2 have the same charges as DDS (except for their missing amine H atoms, of course). Charges for OPO are given assuming that PHE is linked to the left and DD1 or DD2 is linked to the right of the OPO fragment in the figure.

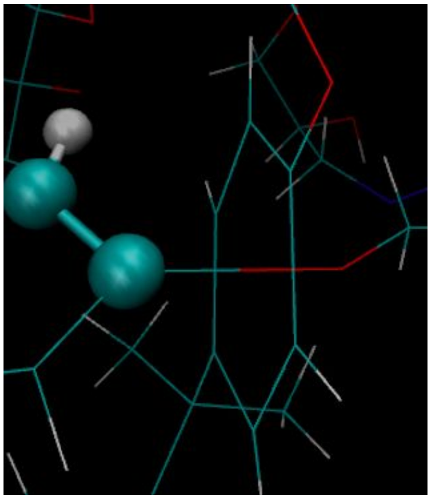

Figure S2. An example of ring spearing observed in the simulations after a smaller interatomic distance than 2.5 Å was used during the system preparation.

2. The Curing Reaction

The possible underlying chemical reaction mechanisms of the curing reaction have been analyzed by Ehlers et al.[1] Applying density functional theory on small model complexes of epoxy and aliphatic amines, they found that the reaction favors a step-wise pathway, which includes the formation of a zwitterionic intermediate IM, over a concerted reaction involving a cyclic transition state (Figure 2a). Both mechanisms can benefit from hydrogen bonding interactions either via unreacted amine hydrogens or even better via the hydroxyl groups of the product species. In line with the analysis by Ehlers et al., we predict similar reaction kinetics for model systems involving primary and secondary aromatic amines as shown in Figure 2b. All structures were fully optimized at the wBP97-xD[2]/def2-TZVP[3] level of theory and characterized by subsequent frequency calculations at the same level of theory. The transition states were further confirmed by IRC calculations.[4,5] Final energies were obtained at the DLPNO-CCSD(T)[6]/def2-QZVPP[7] level of theory with Orca[8]. All optimized structures are summarized in Figure S3 and the respective energetic barriers are stated in Table S1. Furthermore, we estimated the influence of solvent effects on the barrier, which should better reflect the situation in the liquid epoxy mixture (Table S2).

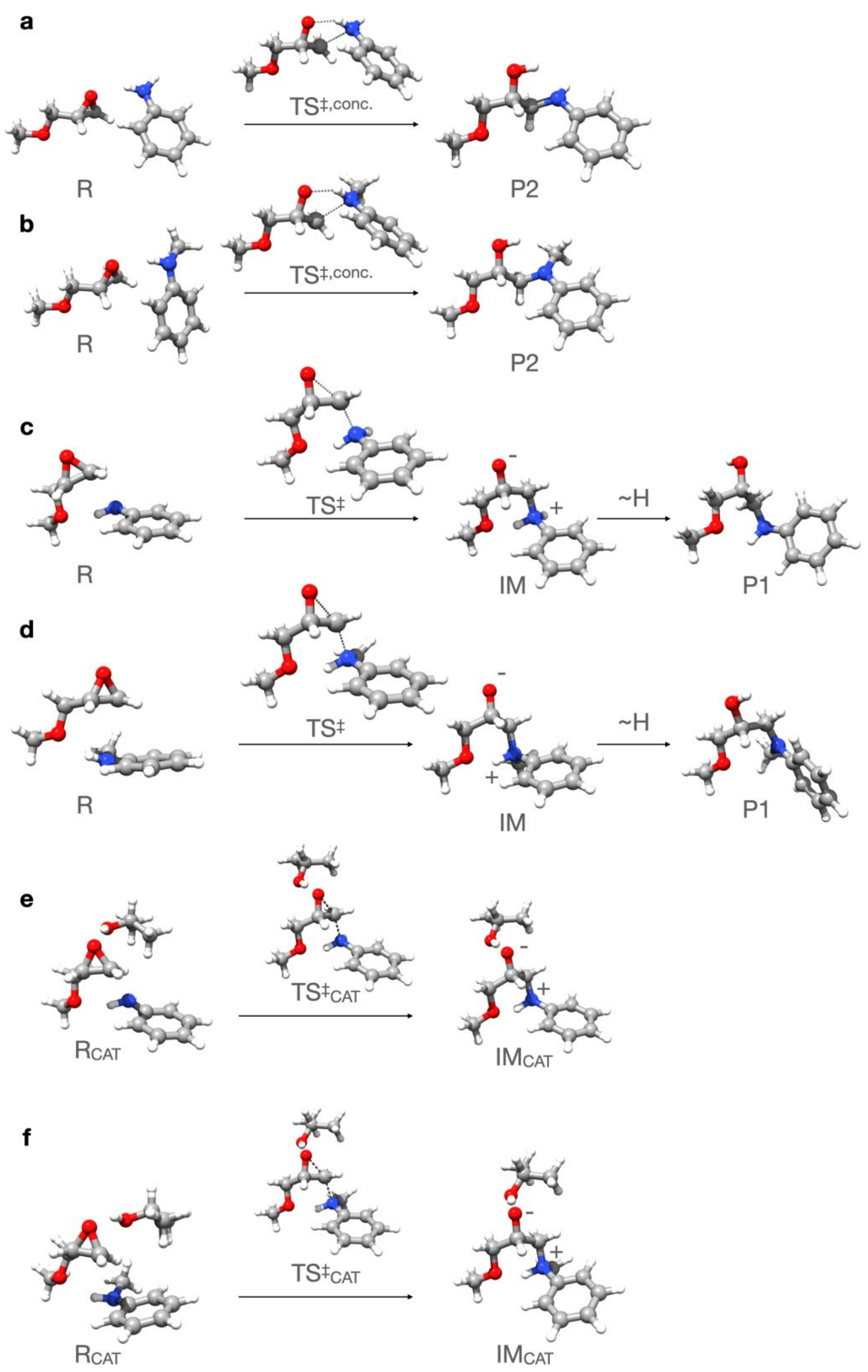

Figure S3. Optimized model reactants, transition states and products for the curing reaction with primary and secondary amines following the scheme in Figure S3. The concerted mechanism is shown in a and b, the step-wise reaction in c and d and the step-wise/alcohol-catalyzed reaction in e and f.

Table S1. Energetic barriers for the first elementary step of the curing reaction with primary and secondary amines depicted in Figure S4.

$System$	$\Delta H^{298.15}[kJ/mol]$ wB97-xD/def2-TZVP[a]	$\Delta H^{298.15}[kJ/mol]$ DLPNO-CCSD(T)/def2-QZVPP[a]
Primary / conc.	213.99	213.01
Secondary / conc.	207.89	204.05
Primary / step-wise	152.33	142.30
Secondary / step-wise	150.82	136.90
Primary / step-wise (cat)	121.28	110.27
Secondary / step-wise (cat)	122.46	106.64

Table S2. Estimation of solvent effects on the barriers for the first elementary step of the alcohol catalyzed curing reaction with primary and secondary amines depicted in Figure S3. Calculations are done at the SMD[9] wB97XD/def2TZVP level of theory. $\Delta\Delta E^{\ddagger}_{solv}$ in kJ/mol.

**solvent**	**Primary**	**Secondary**	**ε**
n-methylaniline	-24.3	-24.3	5.96
Aniline	-28.4	-27.4	6.8882
tetrahydrofuran	-20.6	-21.5	7.4257
1-butanol	-38.7	-35.9	17.332
2-propanol	-36.8	-34.7	19.264
Ethanol	-39.9	-37.1	24.852
methanol	-44.0	-40.2	32.613
1-2-ethanediol	-44.5	-38.1	40.245

3. The curing protocol

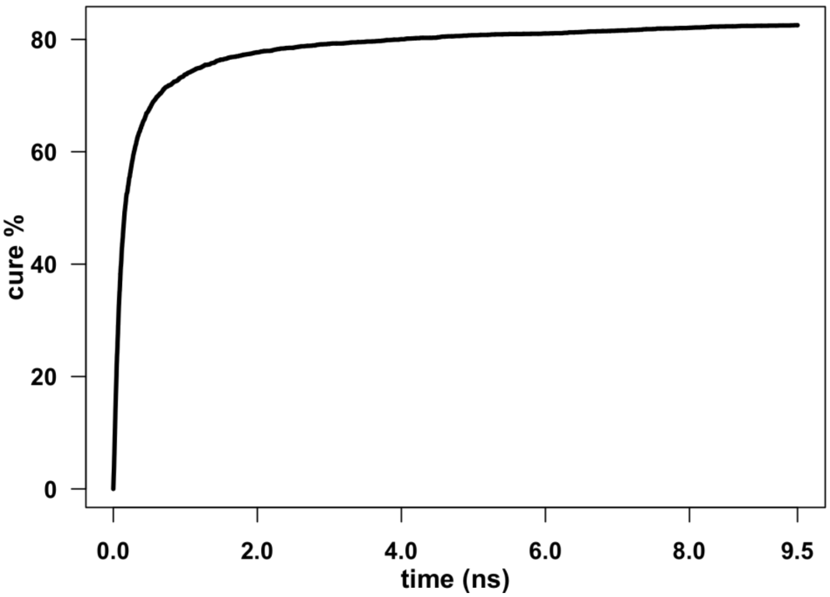

Figure S4. Degree of curing over 9.5 ns for one of our systems, when no annealing is applied. Samples collected every 100 fs.

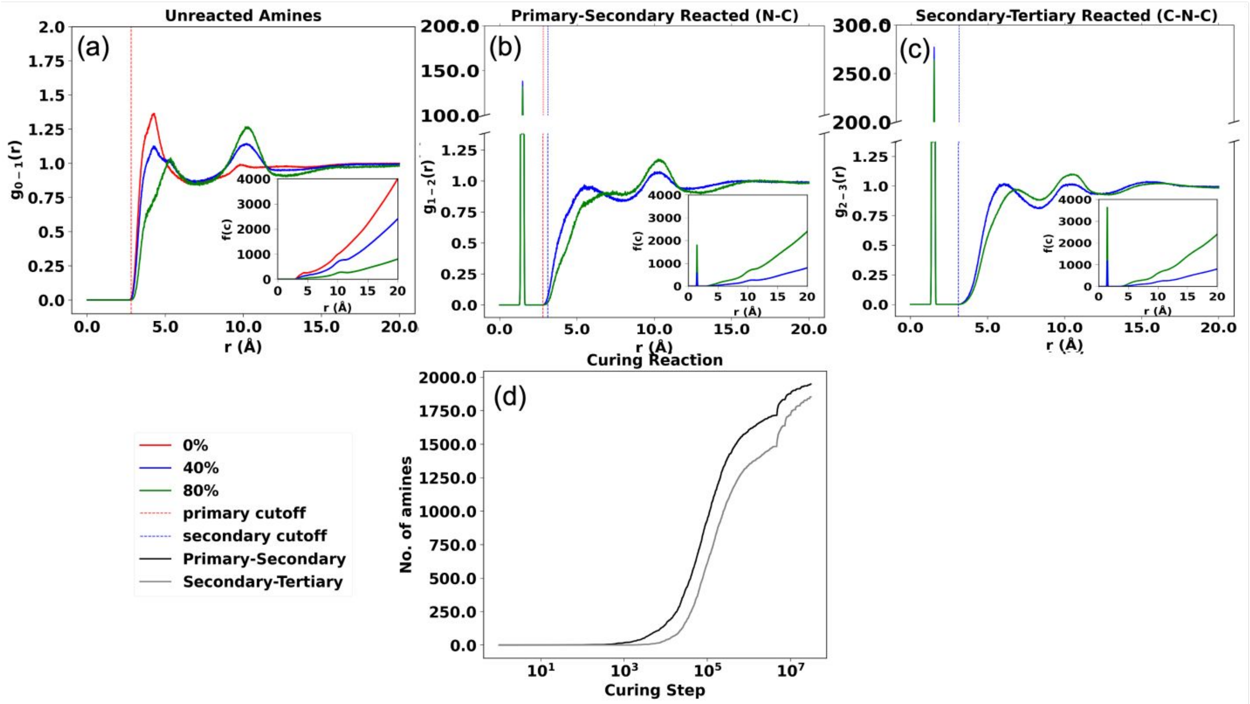

Figure S5. The analysis of the effect of cut-offs on the distribution of reactive groups in the system as a function of curing. Cut-offs are presented with dashed red and blue vertical lines. Radial distribution functions at 0% (red lines), 40% (blue lines), and 80% (green lines) between reactive C and a) primary, b) secondary, and c) tertiary amines are shown. The insets represent the number of reactive C atoms as a function of the separation from the corresponding amine type. d) Number of amine conversion during primary to secondary reaction (black line) and secondary to tertiary reaction (gray line) as a function of curing steps. No secondary amine is discovered up until the primary cutoff (2.8 A) and no tertiary amine are seen up until the secondary cutoff of 3.1 A (Figure 1c). Furthermore, the conversion of primary amines clearly preceeds the conversion of secondary ones.

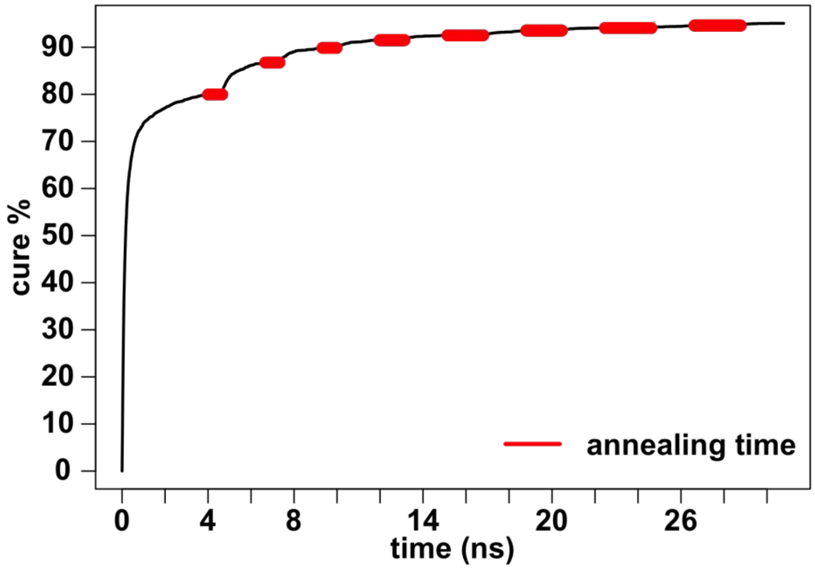

Figure S6. Evolution of the curing extent as a function of time for one of the five epoxy model systems. The slope of the curing curve decreases considerably after the first 4 ns of simulated cross-linking, due to a corresponding decrease in the diffusivity of the reactants. It rises again, however, after an unreactive annealing period at 800 K (marked in red). As the curing extent approaches 90%, longer annealing cycles are needed to obtain an appreciable increase in reaction rate.

4. Equilibration of the epoxy network.

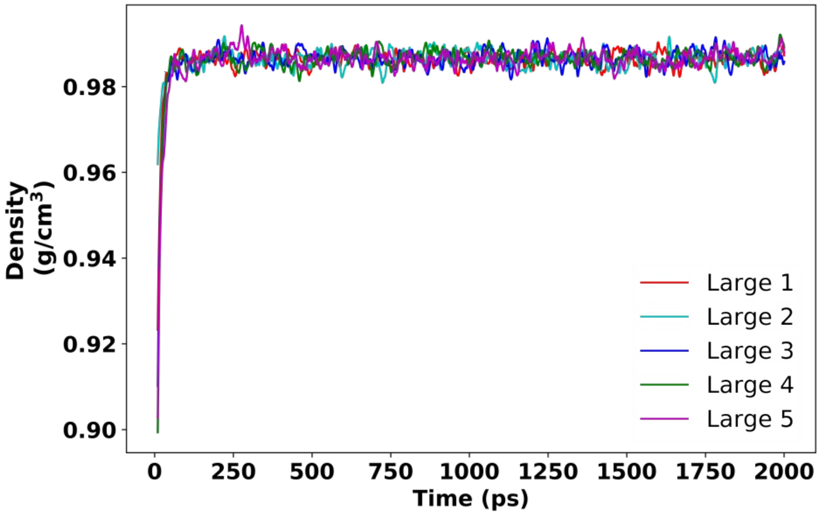

Figure S7. Initial equilibration of the liquid precursor before curing, for all five large systems. The density equilibrated within 250 ps.

Our curing procedure intersperses 100-ps equilibration periods among actual cross-linking cycles. We also performed curing on a large system with twice-as-long equilibration intervals of 200 ps (Figure S11). It can be readily seen that the differences between the 100 and the 200 ps profiles are negligible, indicating that 100 ps is a sufficient, as well as affordable, equilibration time (the disparity between the final density values associated with the two curves is of 0.8%).

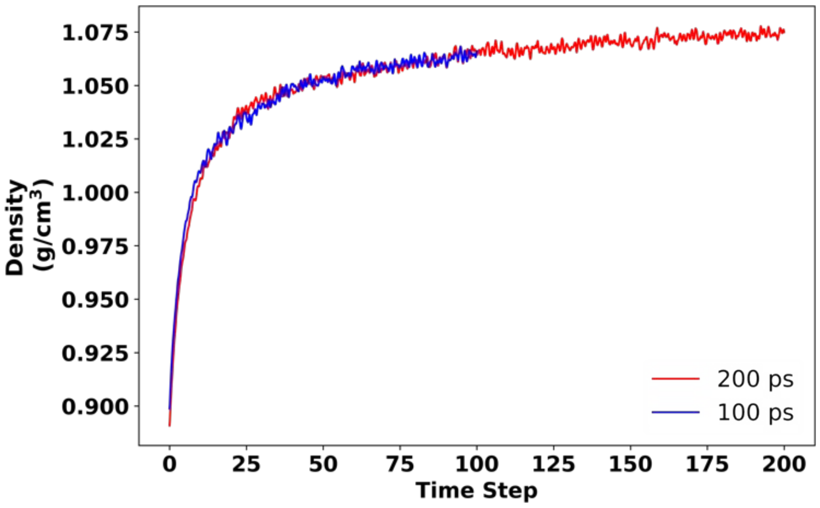

Figure S8. Comparison of the density profiles measured by running 100 ps (blue) and 200 ps (red) NPT equilibration periods after a curing cycle.

For such longer equilibrated system, we detected the point of percolation at 63% conversion, in accordance with what we measured using 100 ps (61 ± 2 %). Likewise, the glass transition temperature remained unaffected by this longer equilibration time at both 35 % (100ps: (78 ± 11)˚C; 200 ps: (79 ± 11)˚C) and 75 % curing (100 ps: (145 ± 11)˚C; 200 ps: (154 ± 11)˚C).

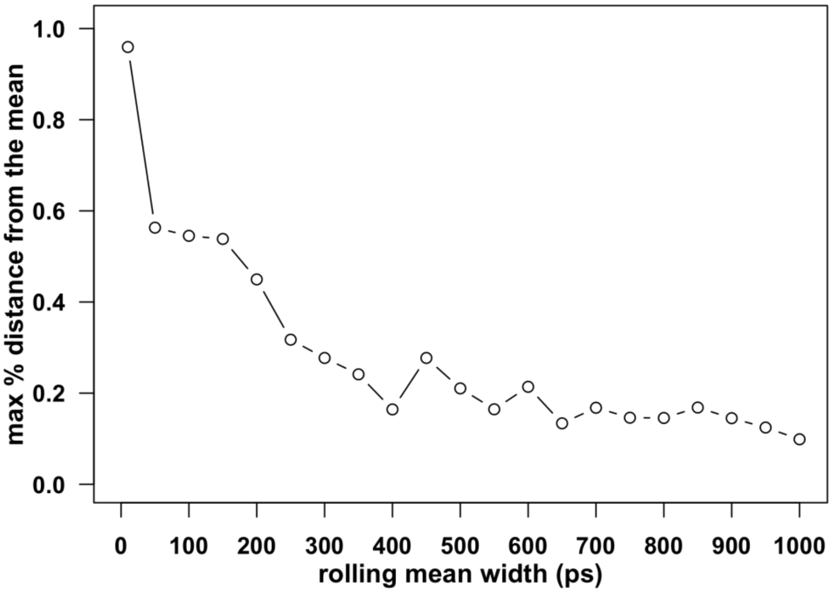

Figure S9. NPT equilibration of the same system as in Figure S4, 75% cured, at 503 K. Maximum % distance from the overall 10-ns average density value as a function of the time width of the window of observation. This shows that after 100 ps, the deviation from the average equilibrium density drops below 1%. The average density was evaluated after a 10 ns NPT run applied to one 75 % cured large system. The graph was built by sampling the density over a set of observation windows of increasing duration, from 100 to 1000 ps, and calculating for each of them the maximum deviation from the mean. This suggests that 100 ps are indeed sufficient for equilibration during curing.

5. Computational efficiency of fix bond/react

Table S3. Benchmarking results for an example crosslinking simulation using fix bond/react and a standard NPT equilibration run. Both simulations were run at 503 K for 1 ns and otherwise identical setups. All simulations were run on the Meggie cluster at the FAU, with each node equipped with two Intel Xeon E5-2630v4 "Broadwell" processors (10 cores per chip).

No.of nodes used	*Fix Bond/React (ns/day)*	*NPT equilibriation (ns/day)*
4	1.932	5.480
8	3.506	9.924
12	4.939	13.986

6. Characterisation of the epoxy network

Table S4. The parameters a, b and c used to rescale each density data set onto the mean 95% plot, the temperature $T_{95\%}$ corresponding to the center of the hyperbola fitted to the mean 95% plot, and the resulting values of glass transition temperature T_g, as a function of curing percentage.

$\boldsymbol{curing}$ %	$\boldsymbol{a}$	$\boldsymbol{b}\ (\boldsymbol{C})$	$\boldsymbol{c}\ (\frac{\boldsymbol{g}}{\boldsymbol{cm^3}})$	$\boldsymbol{T_{hyperbola\ center\ 95\%}}$	$\boldsymbol{T_g}\ (\boldsymbol{C})^{a}$
**5**	1.58 ± 0.03	-45 ± 4	-0.030 ± 0.002		42 ± 10
**15**	1.52 ± 0.04	-40 ± 6	-0.030 ± 0.003		51 ± 11
**25**	1.45 ± 0.02	-30 ± 5	-0.027 ± 0.003		60 ± 10
**35**	1.43 ± 0.04	-50 ± 6	-0.025 ± 0.002		78 ± 11
**45**	1.35 ± 0.03	-37 ± 5	-0.025 ± 0.003	179 ± 11	89 ± 12
**55**	1.31 ± 0.02	-50 ± 5	-0.018 ± 0.002		110 ± 11
**65**	1.27 ± 0.02	-60 ± 6	-0.013 ± 0.002		130 ± 12
**75**	1.20 ± 0.01	-50 ± 6	-0.010 ± 0.002		145 ± 11
**85**	1.10 ± 0.01	-15 ± 4	-0.002 ± 0.002		165 ± 12
95	1.00 ± 0.00	0 ± 0	0 ± 0		179 ± 11

(a) T_g was obtained by inverting the linear transformation on the temperature axis as $T_g = \frac{T_{95\%} - b}{a}$.

Uncertainties on a and b were obtained by visually estimating which set of scaling parameters

yielded an acceptable overlay onto the 95% mean plot, keeping into account the standard deviation in density at each temperature data point. The uncertainty on $T_{95\%}$ represents a 95% confidence interval for the center of the hyperbola fitted to the mean 95%-curing plot. Finally, the uncertainty on T_g was computed by error propagation; the greatest contribution to the error on T_g comes from the estimated uncertainties on the b parameters.

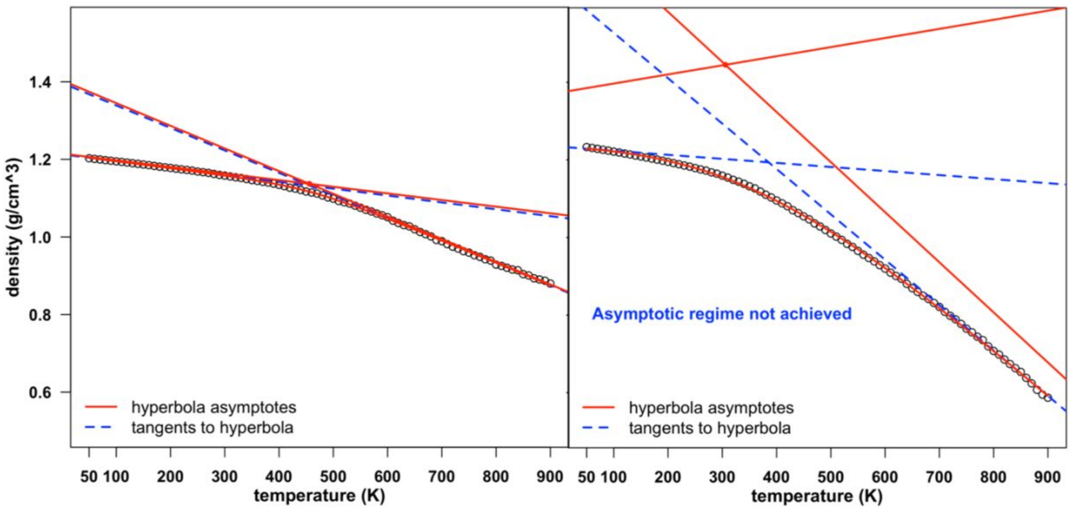

Figure S10. Density as a function of temperature at 95% (left) and at 15% (right) curing extents. The 95% data set shows an excellent hyperbolic trend, as confirmed by the overlap of the hyperbola asymptotes with the analytic tangents to the lowest and highest temperature points of the hyperbola. Conversely, the 15% data set clearly deviates from any hyperbolic trend, as testified by the disagreement between asymptotes and tangents. In this case, neither the intersection of the analytic tangents nor the center of the hyperbola are reliable estimates of T_g.

7. Young's modulus

Table S5. All simulation parameters and results regarding post-curing annealing, equilibration and uniaxial tensile test.

$\boldsymbol{System}$	$\boldsymbol{Annealing\ time\ (ns)}$	$\boldsymbol{Equilibration\ time\ (ns)}$	$\boldsymbol{Uniaxial\ strain\ rate\ (s^{-1})}$	$\boldsymbol{Young's\ modulus}$[a] $\boldsymbol{(GPa)}$
**1**		125		
**2**	10	150	$2\ 10^{-8}$	3.176 ± 0.002
**3**		150		

**4**	150
**5**	100

(a) average over all simulations up to 0.02 strain.

8. Impact of partial atomic charges on curing and physical properties.

To investigate the impact of Coulomb interactions on the cross-linking process and on the deriving physical properties of the cured system, we carried out an additional simulation leaving out all Coulomb interactions (i.e. without charges), but still following the same procedure adopted in the presence of our block-chemistry charges. Specifically, we equilibrated and cured a system of large size (127000 atoms) and then evaluated its glass transition temperature, elastic modulus and percolation point.

8.1 Percolation point

The percolation point of the material cured without partial atomic charges was located at 58%, in alignment with our findings in the presence of charges. This agreement is to be expected, since the Flory-Stockmayer theory of polymer growth can describe gelation solely in terms of the functionalities of the reactants, predicting gelation exactly at 58% in the present case of a tetra-functional hardener (DDS) and a bi-functional resin (DGEBA).

8.2 Glass transition temperature

We investigated samples at 35 % and 75 % curing as examples for curing degrees below and above the gel point. We determined the glass transition temperature using the same analysis procedure as for the block-chemistry approach and found that neglecting charges leads to drastic consequences. Specifically, at 35 % curing, T_g drops from 78 ± 11°C in the system with charges,

to 28 ± 9 ˚C in the one without charges. Similarly, at 75 % curing, the decrease is from 145 ± 11 ˚C to 94 ± 10 ˚C. This is far from the excellent experimental agreement achieved by our block-chemistry approach at all investigated degrees of cross-linking (Figure 4 of the manuscript).

This difference between the systems with and without charges is easy to assess by observing the density as a function of temperature at 35 % and 75 % curing (Fig. 6b in the manuscript). First, the slopes of the low-temperature tangents differ from each other, suggesting a scaling behavior different from the one presented in the manuscript for charged systems. Second, without charges the glass transition region is shifted to lower temperatures. Additionally, the density of the material polymerizing without electrostatic interactions is, at temperatures higher than the transition, systematically lower than the one obtained by using charges.

8.3 Elastic modulus

Finally, we performed in-silico tensile tests on our system cured without charges. By analyzing the linear response region, we determined the Young's modulus to be 2.015 ± 0.002 GPa. This constitutes more than a 30% drop in the ability of the material to withstand tensile stress, compared to the result obtained with charges (Young's modulus of 3.176 ± 0.002 GPa).

9. References

(1) Ehlers, J.-E.; Rondan, N. G.; Huynh, L. K.; Pham, H.; Marks, M.; Truong, T. N. Theoretical Study on Mechanisms of the Epoxy−Amine Curing Reaction. *Macromolecules* **2007**, *40*, 4370–4377.

(2) Chai, J.-D.; Head-Gordon, M. Long-Range Corrected Hybrid Density Functionals with Damped Atom–Atom Dispersion Corrections. *Phys. Chem. Chem. Phys.* **2008**, *10*, 6615–6620.

(3) Weigend, F.; Ahlrichs, R. Balanced Basis Sets of Split Valence, Triple Zeta Valence and Quadruple Zeta Valence Quality for H to Rn: Design and Assessment of Accuracy. *Phys. Chem. Chem. Phys.* **2005**, *7*, 3297–3305.

(4) Page, M.; Doubleday, C.; McIver, J. W. Following Steepest Descent Reaction Paths. The Use of Higher Energy Derivatives with *Ab Initio* Electronic Structure Methods. *J. Chem. Phys.* **1990**, *93*, 5634–5642.

(5) Page, M.; McIver, J. W. On Evaluating the Reaction Path Hamiltonian. *J. Chem. Phys.* **1988**, *88*, 922–935.

(6) Riplinger, C.; Pinski, P.; Becker, U.; Valeev, E. F.; Neese, F. Sparse Maps—A Systematic Infrastructure for Reduced-Scaling Electronic Structure Methods. II. Linear Scaling Domain Based Pair Natural Orbital Coupled Cluster Theory. *J. Chem. Phys.* **2016**, *144*, 024109.

(7) Hellweg, A.; Hättig, C.; Höfener, S.; Klopper, W. Optimized Accurate Auxiliary Basis Sets for RI-MP2 and RI-CC2 Calculations for the Atoms Rb to Rn. *Theor. Chem. Acc.* **2007**, *117*, 587–597.

(8) Neese, F. The ORCA Program System. *Wiley Interdiscip. Rev. Comput. Mol. Sci.* **2012**, *2*, 73–78.

(9) Marenich, A. V.; Cramer, C. J.; Truhlar, D. G. Universal Solvation Model Based on Solute Electron Density and on a Continuum Model of the Solvent Defined by the Bulk Dielectric Constant and Atomic Surface Tensions. *J. Phys. Chem. B* **2009**, *113*, 6378–6396.
